# *Rickettsia parkeri* hypothetical protein RPATATE_1266, a homolog of exopolyphosphatase/guanosine pentaphosphate phosphohydrolase, regulates tick cell apoptosis

**DOI:** 10.1128/spectrum.00151-25

**Published:** 2025-07-07

**Authors:** Dattatray V. Sawant, Nicole Y. Burkhardt, Haritha Katasani, Benjamin Cull, Ulrike G. Munderloh, Xin-Ru Wang

**Affiliations:** 1 Department of Microbiology and Immunology, Upstate Medical University 827267, Syracuse, New York, USA; 2 Department of Entomology, University of Minnesota 311842, St. Paul, Minnesota, USA; 3 SUNY Center for Vector-Borne Diseases, Upstate Medical University 12302, Syracuse, New York, USA; 4 Institute for Global Health and Translational Sciences, Upstate Medical University 12302, Syracuse, New York, USA; National Institutes of Health, Rockville, Maryland, USA

**Keywords:** *Rickettsia parkeri*, apoptosis, RPATATE_1266 mutant rickettsia

## Abstract

*Rickettsia parkeri,* an intracellular bacterium transmitted by *Amblyomma maculatum* ticks, causes a febrile illness associated with eschar formation in humans. As a less virulent member of the spotted fever group within the *Rickettsia* genus, *R. parkeri* serves as an ideal model for studying interactions between rickettsial pathogens, their vectors, and hosts. Our previous research showed that *R. parkeri* modulates mitochondrial-dependent apoptosis in tick cells, enhancing its intracellular survival and replication; however, the underlying mechanisms remain unclear. To investigate further, we employed a Himar1-based transposon mutagenesis system to identify *R. parkeri* genes involved in apoptosis. Using the modified plasmid pLoxHimar, we introduced a transposon into the *R. parkeri* Tate’s Hell strain and selected a mutant with an insertion in RPATATE_1266, a hypothetical protein with homology to exopolyphosphatase/guanosine pentaphosphate phosphohydrolase (Ppx/Gppa) family proteins, using mCherry fluorescence and spectinomycin/streptomycin resistance. The mutant genotype and single insertion site were confirmed by insertion site sequencing and inverse PCR. We found that the RPATATE_1266 mutant had reduced infection rates, growth, and plaque formation compared to wild-type *R. parkeri* in tick and mammalian cells. Moreover, the mutant showed upregulation of genes associated with *ppx/gppa* gene regulation networks (GRNs) and exhibited a marked inhibition of apoptosis in tick cells, including decreased expression of apoptosis-related genes, reduced mitochondrial membrane potential, and less DNA fragmentation. Restoration of RPATATE_1266 resulted in significant recovery of later-phase apoptosis, as measured by Caspase 3/7 activity. These findings suggest that RPATATE_1266 is a critical regulator of apoptosis in tick cells and could be a potential target for controlling *R. parkeri* infection and replication. This research enhances our understanding of how vector responses to pathogen infection influence pathogen replication and transmission.

**IMPORTANCE:**

*Rickettsia parkeri* infections, though less severe than other rickettsioses, are becoming increasingly significant due to the expanding geographic range of their tick vector and their role in shaping our understanding of rickettsial biology. Advancing knowledge of the molecular mechanisms that regulate *R. parkeri* infection and replication is important for the field of vector-pathogen interactions. This study identifies the RPATATE_1266 gene (a hypothetical protein with homology to exopolyphosphatase/guanosine pentaphosphate phosphohydrolase [Ppx/Gppa] family proteins) as a key regulator of mitochondrial-dependent apoptosis in tick cells, a process critical for rickettsial intracellular survival. By elucidating the role of this gene, we provide new insights into the molecular interactions between rickettsial pathogens and their vectors. These findings not only enhance our understanding of pathogen-vector dynamics but also highlight potential directions for developing future strategies to manage rickettsial diseases beyond those caused by *R. parkeri.*

## INTRODUCTION

*Rickettsia parkeri* (genus *Rickettsia*, family *Rickettsiaceae*, order *Rickettsiales*), a tick-borne intracellular bacterium that causes a febrile, eschar-associated illness in humans, was first discovered in the Gulf Coast tick, *Amblyomma maculatum* Koch, in 1937 (1). Its pathogenicity was confirmed in 2002 and has since been described as “*R. parkeri* rickettsiosis.” As a member of the spotted fever group of the genus *Rickettsia*, the *R. parkeri* life cycle is closely related to *Rickettsia rickettsii*, the agent of Rocky Mountain spotted fever, although it causes a less severe rickettsiosis (2, 3). Thus, *R. parkeri* serves as an ideal model for studying rickettsial pathogen-host interactions, and significant progress has been made in this area (4, 5). Despite *R. parkeri*’s dependence on ticks for its life cycle, our understanding of how it manipulates the innate immune systems of its tick vectors to facilitate acquisition, persistence, and transmission remains limited.

Apoptosis, a type I programmed cell death, is a component of innate immunity that can respond to a wide range of pathogens, including tick-borne pathogens like *Anaplasma phagocytophilum*, *R. rickettsii*, and *R. parkeri* (6, 7). For tick-borne pathogens, apoptosis functions not only as a host defense mechanism but also as a pathway that pathogens frequently manipulate to enhance their intracellular survival and replication. For instance, our previous work demonstrated that apoptosis activation in response to *R. parkeri* infection is a conserved response in tick cell lines, with mitochondrion-dependent apoptosis potentially facilitating rickettsial infection and replication. Although rickettsial intracellular replication is essential for apoptosis induction, the specific apoptosis-regulatory molecules (ARMs) produced by *R. parkeri* that target tick cells to trigger apoptosis remain unknown (8). Genetic modification of rickettsiae remains challenging, due to various features of the genus *Rickettsia*, including highly reduced genomes and their obligate intracellular growth requirements; thus, the identity of these ARMs is still a mystery (9–11).

The generation and functional characterization of mutant libraries for rickettsial species have enhanced our understanding of rickettsial pathogen biology. Although recent studies have reported successful transformations of several rickettsial pathogens within the order *Rickettsiales*, including *A. phagocytophilum* (genus *Anaplasma*, family *Anaplasmataceae*), *E. chaffeensis* (genus *Ehrlichia*, family *Anaplasmataceae*), *Rickettsia prowazekii*, *R. rickettsii*, and *R. parkeri* (12), these efforts still lag behind progress made with extracellular bacteria, largely due to the obligate intracellular characteristics of rickettsiae. The Himar1-based transposon system, a powerful tool for random mutagenesis of obligate intracellular bacterial genomes, has been widely used to identify genes associated with bacterial cell cycle and metabolism, pathogenesis, host interactions, and previously unknown factors by generating loss-of-function mutants through transposon insertions (13). For example, a previous collaboration with Arroyave et al. (14) resulted in the establishment of an *R. parkeri* mutant library to explore pathogen-host interactions and pathogenesis in rickettsial species. One mutant (RPATATE_0245::pLoxHimar, named 3A2) harbors a genetically disrupted phage integrase gene. This mutant, with significantly attenuated virulence and strong immunogenicity, demonstrated potent protective efficacy against fatal rickettsioses in a murine model, highlighting its potential as a foundation for multivalent vaccine platforms (14).

Following our identification of apoptosis as an important pathway involved in *R. parkeri* infection of tick cells (8), we screened the mutant library for *R. parkeri* strains with defects in inducing apoptosis. This approach led to the identification of the RPATATE_1266 mutant, which inhibited apoptosis as initially observed in a DNA fragmentation assay (X. Wang, unpublished data), revealing a previously unrecognized role for this gene in regulating apoptosis in tick cells. This unexpected phenotype prompted us to explore the link between this bacterial regulatory gene and host apoptosis. Prior studies—including our own work—have shown that *R. parkeri* can modulate mitochondrial-dependent apoptosis in tick cells, and Ppx/Gppa enzymes that are primarily known for roles in bacterial stress responses and metabolic regulation appear to participate in this process. Given that intracellular pathogens often utilize bacterial regulatory pathways to influence host cell death and survival, we hypothesized that RPATATE_1266 may play a dual role in intracellular adaptation and tick apoptosis modulation. This study, therefore, investigates whether disruption of this gene alters apoptosis-related outcomes during infection. Ppx/Gppa enzymes, including exopolyphosphatase (Ppx) and guanosine pentaphosphate phosphohydrolase (Gppa), are critical in bacterial stress responses and metabolic regulation by degrading polyphosphate and hydrolyzing alarmone molecules like (p)ppGpp, thereby facilitating bacterial adaptation to nutrient deprivation and other environmental stresses (15). In pathogenic bacteria, *ppx/gppa* activity is associated with survival under host-imposed stress and may impact virulence and persistence (16–18). For instance, *Helicobacter pylori*, a stomach pathogen infecting over 50% of the global population and associated with duodenal ulcers and stomach cancer, faces a highly acidic environment, nutrient deprivation, host defenses, and antibiotic exposure. To survive these conditions, *H. pylori* relies on tight regulation of PolyP and (p)ppGpp homeostasis, managed by a single *ppx/gppa* homolog (HpPPX/GppA), in contrast to *Escherichia coli*, which has two homologs, EcGppA and EcPPX (19). Similarly, *Rickettsia* species encode only a single *ppx/gppa* homolog, which may play an important role in maintaining homeostasis and adapting to environmental stress. The conservation of *ppx/gppa* enzymes across the *Rickettsia* genus and their unique presence within this group suggests an evolutionary role in host-pathogen interactions, potentially modulating host cell death pathways. We hypothesize that the *ppx/gppa* homolog *RPATATE_1266* in *R. parkeri* may regulate apoptosis to support intracellular survival. To investigate this, we employed a series of methods, including examining apoptosis-related gene expression, mitochondrial membrane potential, and DNA fragmentation, to validate the apoptotic effects of the RPATATE_1266 mutant. Additionally, we restored the *RPATATE_1266* gene in the mutant strain to further assess its impact on apoptosis in tick cells. Our study aims to reveal the broader role of this pathway in pathogen-vector dynamics and explore the potential of targeting RPATATE_1266 to control *R. parkeri* infection and replication.

## RESULTS

### Characterization of the *R. parkeri* mutant RPATATE_1266 (*Rp*_ Δ1266)

**Fig 1:**
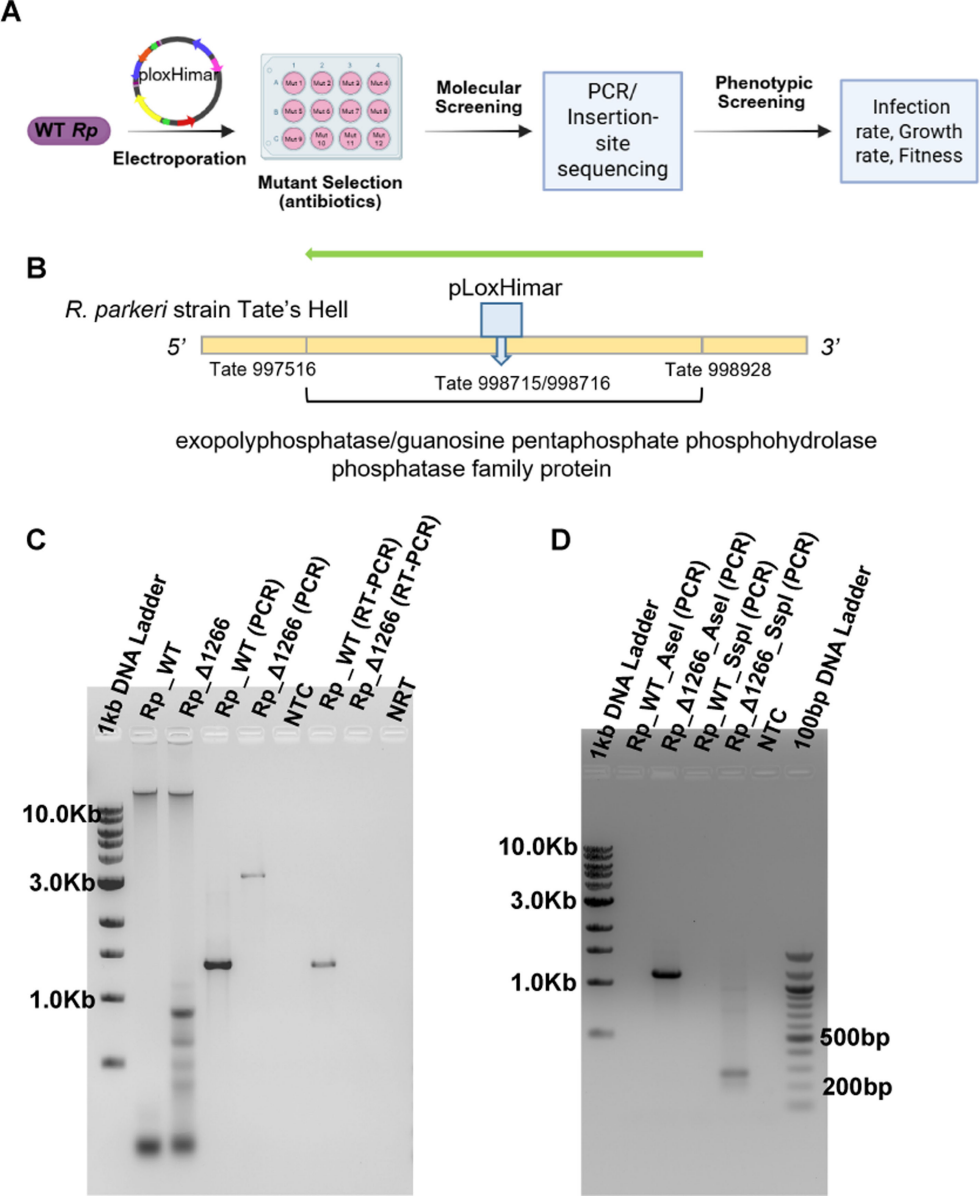
Characterization of the *R. parkeri ppx/gppa* mutant. (**A**) Workflow for the generation of the *R. parkeri RPATATE_1266* mutant strain (*Rp*_Δ1266) using the pLoxHimar transposon system. (**B**) Schematic showing identification of the pLoxHimar transposon insertion site within the RPATATE_1266 locus (Tate_997516 to Tate_998928) by sequencing. The PCR product is indicated by the green arrow. The expected product size is 1,432 bp in *Rp_*WT and 3,327 bp in *Rp*_Δ1266. (**C**) Confirmation of the insertion site by PCR and RT-PCR using primers flanking the RPATATE_1266 locus. Lane 1: DNA ladder; Lane 2: genomic DNA from *Rp*_WT; Lane 3: genomic DNA from RPATATE_1266 mutant (*Rp*_Δ1266); Lane 4: PCR product amplified from WT gDNA (1,432 bp); Lane 5: PCR product amplified from mutant gDNA (3,327 bp); Lane 6: negative control; Lane 7: RT-PCR product from WT RNA (1,432 bp); Lane 8: RT-PCR product from mutant RNA; Lane 9: negative control. Representative results are shown. (**D**) Confirmation of the single Himar transposon insertion site by inverse PCR. Lane 1: 1 kb DNA ladder; Lane 2: PCR product of genomic DNA from *Rp*_WT digested with restriction enzyme AseI and fragment ligated; Lane 3: PCR product of genomic DNA from *Rp*_Δ1266 digested with restriction enzyme AseI and fragment ligated (1,165 bp); Lane 4: PCR product of genomic DNA from *Rp*_WT digested with restriction enzyme SspI and fragment ligated; Lane 5: PCR product of genomic DNA from *Rp*_Δ1266 digested with restriction enzyme SspI and fragment ligated (364 bp); Lane 6: negative control; Lane 7: 100 bp ladder. Specific amplification products corresponding to genomic regions adjacent to the insertion were detected, verifying the presence of a single integration event.

We generated an *R. parkeri* mutant strain by inserting the pCis Himar Cherry A7 Lox (pLoxHimar) into TA dinucleotide sequences within the *R. parkeri* genome, as illustrated in the workflow in Fig. 1A. The pLoxHimar plasmid contains a transposon encoding mCherry/aadA, which allowed mutant selection through spectinomycin/streptomycin resistance and visualization of the red fluorescent marker (mCherry) using fluorescence microscopy. Sequencing of positive clones confirmed that the pLoxHimar transposon was inserted at nucleotide position 998715/998716 of the *R. parkeri* strain Tate’s Hell genome (GCA_000965145.1), disrupting the open reading frame of a gene encoding a homolog to the exopolyphosphatase/guanosine pentaphosphate phosphohydrolase (Ppx/Gppa) family protein (Tate 997516–Tate 998928; Fig. 1B). To further verify the insertion, we designed primers flanking the RPATATE_1266 locus, and PCR showed that amplification of the *RPATATE_1266* gene resulted in a 3,327 bp product for *Rp*_ Δ1266 strain and a 1,432 bp product for the wild-type *R. parkeri* (*Rp*_WT), confirming the presence of the transposon insertion in the mutant. No amplification was observed in reverse transcription polymerase chain reaction (RT-PCR) analysis of *Rp*_ Δ1266, suggesting that the insertion disrupted transcription of the *ppx/gppa* open reading frame (Fig. 1C). To verify the specific insertion site of the Himar transposon in the RPATATE_1266 locus, we performed a modified inverse PCR assay (described in detail in the Materials and Methods). Primers were designed to amplify genomic regions flanking the transposon insertion site. This approach produced specific amplification products corresponding to sequences adjacent to the transposon, confirming a single insertion event (Fig. 1D). These results confirmed the successful generation of an *R. parkeri* RPATATE_1266 mutant strain, providing a foundation for further functional analyses.

### Comparative analysis of Ppx/Gppa

To understand the evolutionary relationship of the Ppx/Gppa protein in *R. parkeri*, we performed a phylogenetic analysis across 38 bacterial species (Table S1). An unrooted phylogenetic tree was constructed using the maximum likelihood method with 1000 bootstrap replications in PhyML-3.1 software (Fig. S1). This revealed that *R. parkeri* Ppx/Gppa clustered closely with those of other *Rickettsia* species, including *Rickettsia typhi*, *R. prowazekii*, and *R. rickettsii*, forming a distinct clade. This clustering highlights the evolutionary conservation of Ppx/Gppa within the *Rickettsia* genus, compared with its divergence from more distantly related bacterial genera such as *Escherichia*, *Salmonella*, and *Helicobacter*. Interestingly, only a single *ppx/gppa* gene was identified in *Rickettsia* species, unlike *E. coli*, which possesses both *ppx* and *gppa* genes, reflecting distinct evolutionary and functional adaptations among these bacteria. We further performed a multiple sequence alignment of Ppx/Gppa sequences from selected bacterial species, including *Rickettsia* species (*R. typhi*, *R. prowazekii*, *R. parkeri*, and *R. rickettsii*), *E. coli*, and *Helicobacter pylori* (Fig. S2) to explore the conservation of Ppx/Gppa. The alignment revealed a high degree of conservation among the *Rickettsia* species, particularly within domains associated with the sugar kinase/actin/HSP70 superfamily, highlighted by red lines. Conserved residues, highlighted by darker shading, showed the structural stability and functional importance of this protein across diverse bacterial species. Together, these results demonstrated that *Rickettsia* species possess a single, highly conserved *ppx/gppa* gene, further supporting its role as a functionally critical protein and a potential target for future studies.

### Comparison of phenotypic characteristics between *Rp*_WT and *Rp*_Δ1266

**Fig 2:**
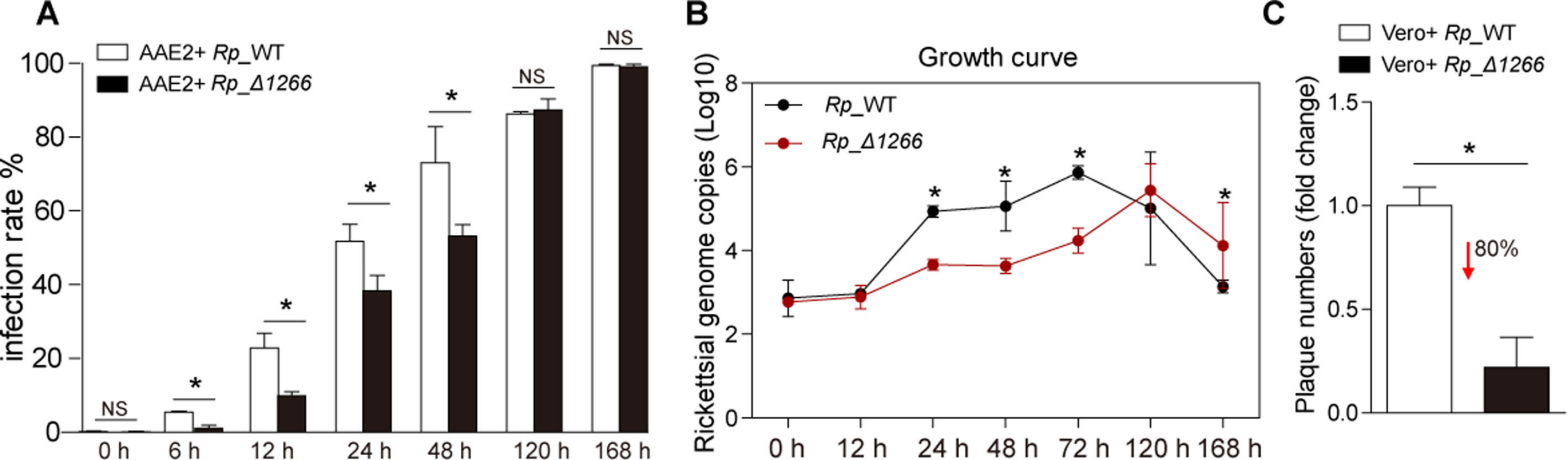
Comparative analysis of infection, growth, and virulence between *Rp*_WT and *Rp_*Δ1266. (**A**) Infection rate (%) of AAE2 cells infected with WT_*Rp* and *Rp_*Δ*1266* mutant at various time points (0, 6, 12, 24, 48, 120, and 168 hours post-infection). Giemsa staining was used to determine the infection rate (ratio of *Rickettsia*-positive cells to total cells) across different fields. (**B**) Growth curves for *Rp*_WT and *Rp_*Δ1266 in AAE2 cells, measured by quantifying *Rickettsia* genomic DNA using the single-copy *gltA* gene to determine rickettsial copy number through qPCR. Data are presented as log10 *gltA* copies over time (0 to 168 hours). Asterisks indicate significant differences between *Rp*_WT and *Rp_*Δ1266 at specific time points (*P* < 0.05). (**C**) Plaque assay at 4 days post-infection. Plaque numbers (fold change) in Vero cells infected with *Rp*_WT and *Rp_*Δ1266 mutant strains. Plaque formation by *Rp_*Δ1266 was reduced by approximately 80% compared to *Rp*_WT. In panels A (analysis of variance [ANOVA] followed by Bonferroni test), B, and C (Student’s two-tailed *t*-test), data are presented as mean ± SD, with * above the columns indicating significant differences.

To investigate the phenotypic impact of the *RPATATE_1266* mutation in *R. parkeri*, we compared infection rate, growth, and plaque formation between the *Rp*_WT and *Rp_*Δ1266. First, the infection rates of *Rp*_WT and *Rp_*Δ1266 in AAE2 cells were assessed at multiple time points post-infection (0, 6, 12, 24, 48, 120, and 168 hours) using Giemsa staining. *Rp*_WT showed a higher infection rate in AAE2 cells compared to *Rp_*Δ1266, with significant differences observed at earlier time points (6, 12, 24, and 48 hours) (Fig. 2A). However, at later stages (120 and 168 hours post-infection), no statistically significant differences were observed. Second, the growth curve was analyzed by quantifying *R. parkeri* genomic DNA using the single-copy *gltA* gene to determine rickettsial copy numbers through quantitative PCR (qPCR). *Rp_*Δ1266 mutant exhibited delayed replication and a significantly reduced growth rate at earlier time points, as reflected in the lower *gltA* copy numbers compared to *Rp*_WT at multiple time points. However, by 120 hours post-infection, the *Rp_*Δ1266 mutant and *Rp*_WT showed comparable growth rates (Fig. 2B). Finally, a plaque assay in Vero cells was used to compare the difference in reduced plaque formation between wild-type *R. parkeri* and the mutant. Plaque formation by *Rp_*Δ1266 showed a significant reduction, with plaque numbers approximately 80% lower than in *Rp*_WT at 4 days post-infection, suggesting markedly impaired cell-to-cell spread and intracellular replication (Fig. 2C). Together, these results demonstrated that the disruption of RPATATE_1266 significantly impacts the infection dynamics, growth, and overall fitness of *R. parkeri* in both tick and mammalian cells.

### Regulatory role of *ppx/gppa* in gene expression

**Fig 3:**
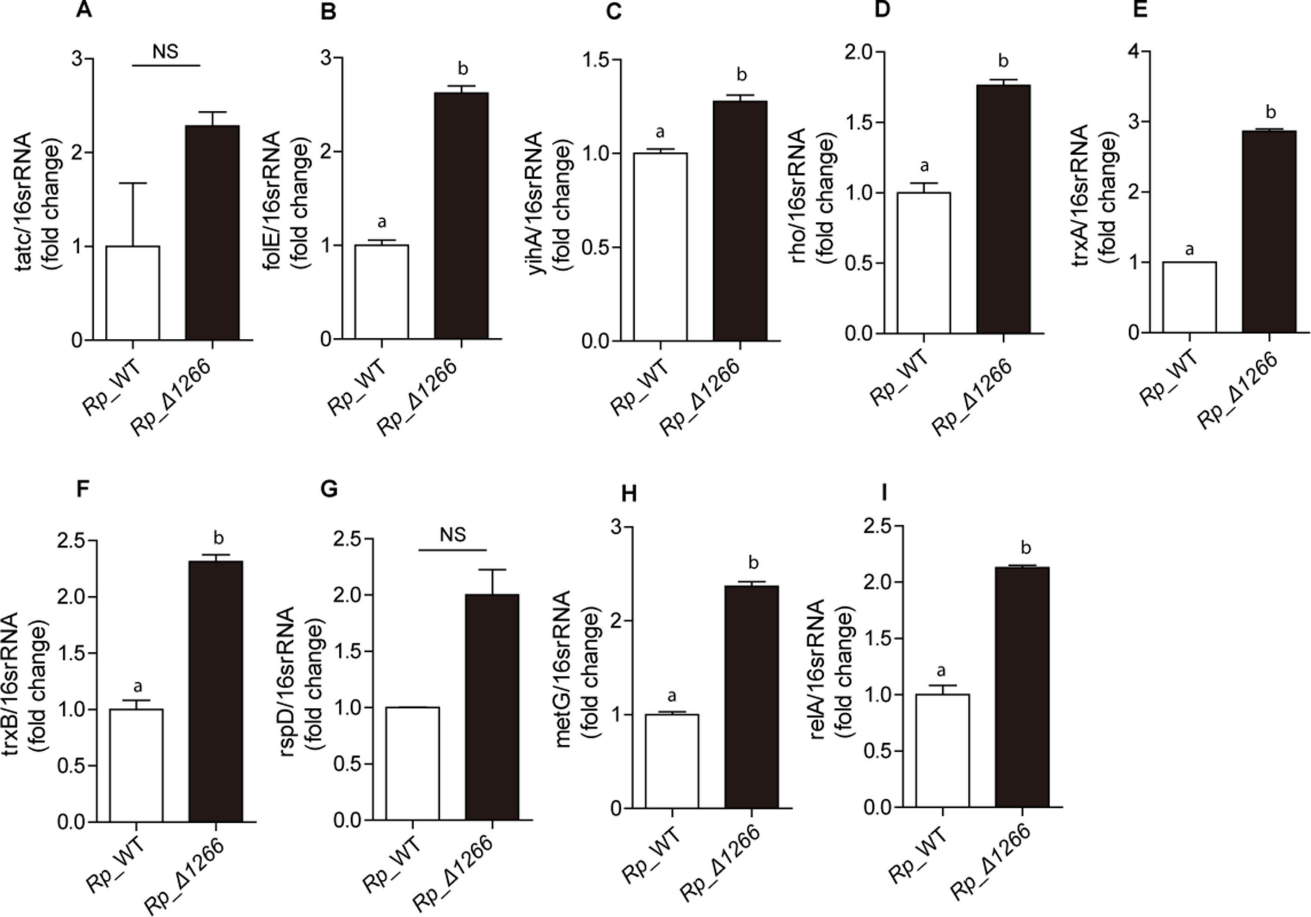
*RPATATE_1266*-regulated gene expression. The relative expression levels of genes associated with *R. parkeri*’s *RPATATE_1266* were analyzed by qRT-PCR in *Rp*_WT and *Rp_*Δ1266, with 16S rRNA expression used as the internal control. The genes examined included (**A**) *tatC*, (**B**) *folE*, (**C**) *yihA*, (**D**) *rho*, (**E**) *trxA*, (**F**) *trxB*, (**G**) *rpsD*, (**H**) *metG*, and (**I**) *relA*. Data are presented as mean ± SD, with different letters above the columns indicating significant differences. Relative expression levels were calculated and analyzed using Student’s two-tailed *t*-test.

The *ppx/gppa* gene, known for its role in (p)ppGpp metabolism and stringent response regulation, plays a key role in transcriptional networks associated with bacterial stress response, metabolic processes, and intracellular survival (20, 21). To explore the regulatory role of RPATATE_1266 in *R. parkeri*, we used the STRING database (22) to identify genes functionally associated with Ppx/GppA in other intracellular bacteria. We then searched for homologs of these genes in the *R. parkeri* genome and analyzed their transcriptional profiles in the *Rp_*Δ1266 mutant strain. Quantitative real-time reverse transcription polymerase chain reaction (qRT-PCR) analysis of nine selected genes exhibited significant differences in expression between WT_*Rp* and the *Rp_*Δ1266 mutant (Fig. 3A through I). These genes included *tatC* (twin-arginine translocation pathway component), *folE* (GTP cyclohydrolase I), *yihA* (GTP-binding protein), *rho* (transcription termination factor), *trxA* (thioredoxin), *trxB* (thioredoxin-disulfide reductase), *rpsD* (30S ribosomal protein S4), *metG* (methionine-tRNA ligase), and *relA* (bifunctional (p)ppGpp synthetase/hydrolase). Most genes, including *folE*, *yihA*, *rho*, *metG*, *relA*, *trxA*, and *trxB*, were significantly upregulated in the mutant, while *tatC* and *rpsD* showed no significant changes in expression compared to wild-type *R. parkeri*, indicating adaptive responses. The transcriptional changes likely result from altered (p)ppGpp levels, which disrupt cellular homeostasis and trigger compensatory transcriptional shifts. Together, the results suggested that the proposed *ppx/gppa* in the RPATATE_1266 mutant serves as a regulatory hub, modulating stress responses, metabolic pathways, and virulence-related genes, thereby impacting transcriptional networks critical for *R. parkeri* survival and pathogenesis.

### Mutation of RPATATE_1266 restricts apoptosis activated by *R. parkeri*

**Fig 4:**
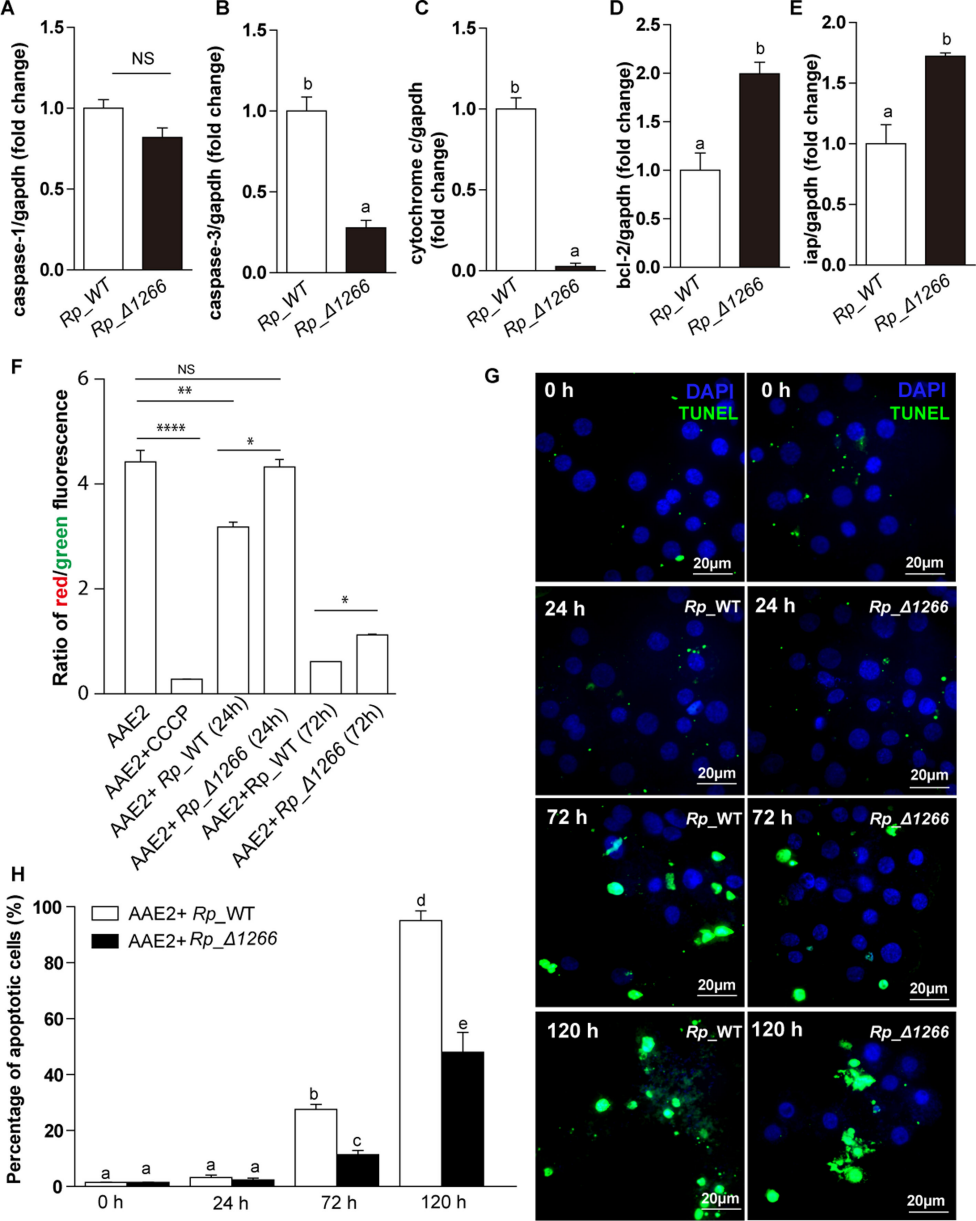
Mutation of *RPATATE_1266* restricts apoptosis activated by *R. parkeri* infection. (**A through E**) The relative expression levels of apoptosis-related genes were analyzed by qRT-PCR in *Rp*_WT-infected and *Rp_*Δ1266-infected AAE2 cells, with *gapdh* expression used as the internal control. (**F**) Mitochondrial membrane depolarization was assessed in AAE2 cells infected with *Rp*_WT and *Rp_*Δ1266 at 24 and 72 hours post-infection, using JC-1 staining. The red/green fluorescence intensity ratio reflects mitochondrial membrane polarization status (higher ratio = healthy mitochondria; lower ratio = depolarization). Carbonyl cyanide 3-chlorophenylhydrazone (CCCP) treatment served as a positive control for complete mitochondrial depolarization. Statistical comparisons were made between *Rp*_WT and *Rp*_Δ1266 at each corresponding time point. Statistical significance was determined by one-way ANOVA followed by Bonferroni post hoc test: ****P < 0.0001; **P < 0.01; *P < 0.05; NS, not significant. (**G**) AAE2 cells were fixed and labeled with Terminal deoxynucleotidyl transferase dUTP Nick End Labeling (TUNEL) (green). Scale bar: 20 µm. DAPI staining (blue) marks the nuclei. (**H**) Quantification of apoptotic cells (TUNEL-positive/DAPI-positive cells) across different fields. In panels A–E (Student’s two-tailed *t*-test), F and G (ANOVA followed by Bonferroni test), data are presented as mean ± SD, with different letters above the columns indicating significant differences.

To investigate the impact of the RPATATE_1266 mutation on apoptosis during *R. parkeri* infection in tick cells, we first examined the expression levels of five apoptosis-related genes: *caspase-1*, *caspase-3*, *cytochrome c*, *bcl-2* (B-cell lymphoma-2), and *iap* (inhibitor of apoptosis protein), which are established markers for studying apoptosis in tick cells (8). Real-time PCR results revealed that in *Rp_*Δ1266*-*infected cells, *caspase-3* and *cytochrome c* expression levels were significantly downregulated, while the anti-apoptotic genes *bcl-2* and *iap* were significantly upregulated. In contrast, *Rp*_WT-infected cells exhibited increased expression of pro-apoptotic genes (*caspase-3* and *cytochrome c*) and no significant change in *caspase-1* expression, indicating that the mutation in RPATATE_1266 dampens the apoptotic response triggered by *R. parkeri* infection (Fig. 4A through E).

To further assess the apoptosis response in *Rp_*Δ1266 *and Rp*_WT-infected cells, we evaluated mitochondrial membrane potential (ΔΨm) changes using JC-1, a cationic carbocyanine dye previously identified as an effective marker of tick cell apoptosis (8). Quantification of the red/green fluorescence intensity ratio showed a significant reduction in *Rp*_WT-infected cells at 24 hours post-infection, indicating mitochondrial dysfunction. In contrast, *Rp_*Δ1266-infected cells maintained a higher red/green fluorescence ratio, similar to uninfected controls. Interestingly, at 72 hours post-infection, *Rp_*Δ1266-infected cells exhibited a reduced red/green fluorescence ratio compared to 24 hours, suggesting that mitochondrial depolarization occurred during prolonged infection. Meanwhile, in *Rp*_WT-infected cells, the red/green fluorescence ratio was significantly reduced, comparable to cells treated with carbonyl cyanide 3-chlorophenylhydrazone (CCCP), a known inducer of mitochondrial depolarization (Fig. 4F). These findings suggest that the *RPATATE_1266* mutation delays apoptosis during early infection stages, but mitochondrial dysfunction may still occur over time in *Rp_*Δ1266-infected cells.

To further verify the activation of apoptosis in *Rp_*Δ1266 *and Rp*_WT-infected cells, we performed a TUNEL assay to detect DNA fragmentation. *Rp_*Δ1266-infected cells showed significantly fewer TUNEL-positive cells, indicating reduced apoptosis (Fig. 4G), while *Rp*_WT-infected cells exhibited high levels of apoptosis, consistent with our previous study (8). Quantification of TUNEL-positive/DAPI-positive cells across different fields further confirmed a marked decrease in apoptotic cells in *Rp_*Δ1266-infected samples compared to WT_*Rp*-infected samples (Fig. 4H), even at 120 hours post-infection, when both showed similar infection growth rates. This indicates that the *RPATATE_1266* mutation causes a defect in apoptosis and that the reduced apoptosis observed is not a result of delayed infection by the mutant. To further confirm that this reduced apoptosis was specific to the *RPATATE_1266* mutation, we analyzed apoptosis in AAE2 cells infected with a control mutant strain carrying an intergenic insertion (Fig. S3). TUNEL assay results revealed apoptotic rates similar to WT_*Rp*-infected cells. These findings indicate that the intergenic insertion mutation method itself does not affect the apoptotic response, further supporting the role of *RPATATE_1266* in modulating apoptosis during *R. parkeri* infection. Together, these results demonstrate that the *RPATATE_1266* mutation restricts apoptosis during *R. parkeri* infection by modulating the expression of apoptosis-related genes, preserving mitochondrial membrane potential, and reducing DNA fragmentation. This emphasizes the critical role of *RPATATE_1266* in mediating apoptosis during *R. parkeri* infection.

### Restoration of *RPATATE_1266* in *R. parkeri* and its effect on apoptosis recovery

**Fig 5:**
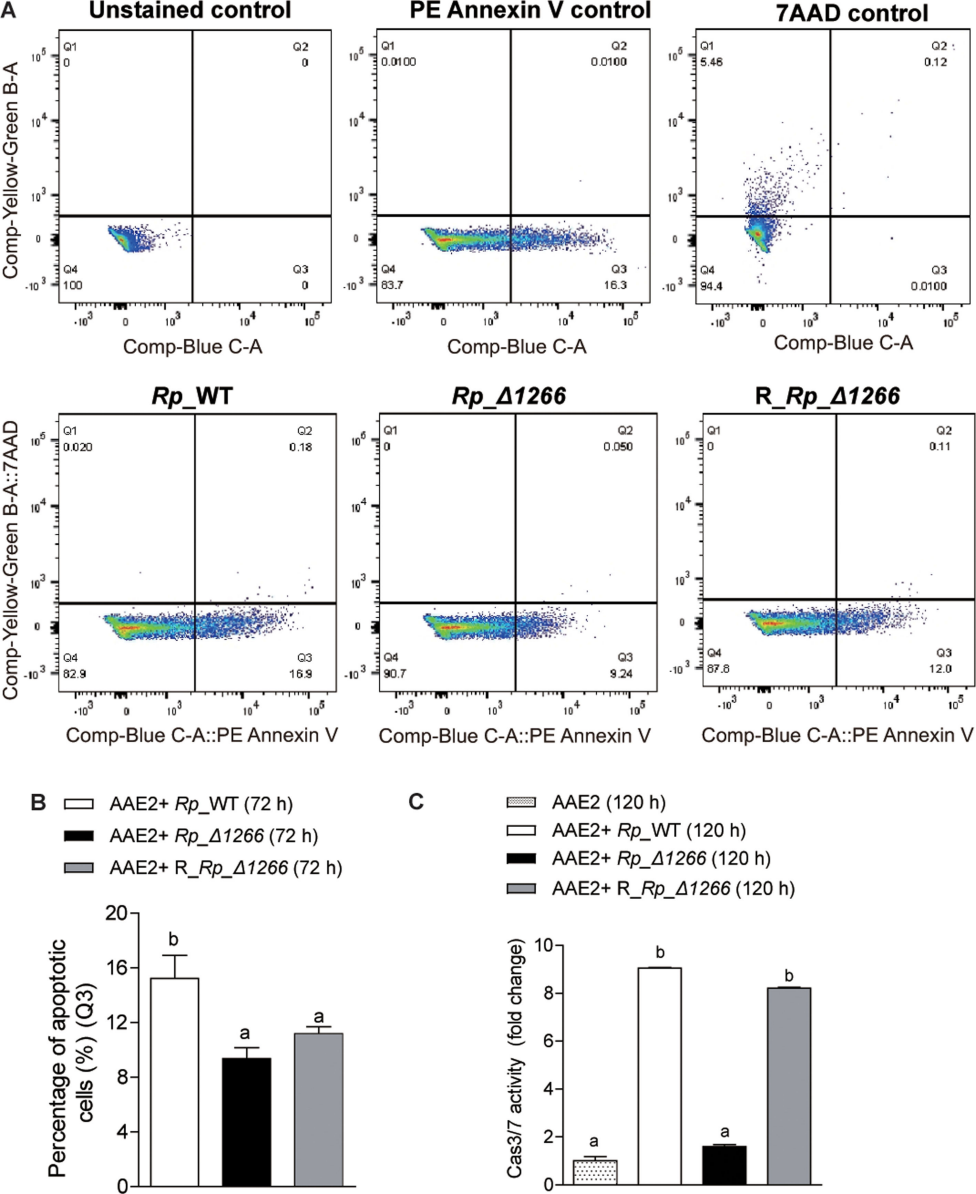
Restoration of *RPATATE_1266* in *R. parkeri* and its effect on apoptosis recovery. (**A**) Flow cytometry analysis of apoptosis during early infection (72 hours post-infection) in *Rp*_WT, *Rp_*Δ1266, and R_*Rp_*Δ1266-infected AAE2 cells. Cells were stained with PE Annexin V to label PS exposure and 7-amino-actinomycin (7-AAD) to distinguish early apoptotic (PE Annexin V positive, 7-AAD negative) from late apoptotic or necrotic cells (PE Annexin V and 7-AAD positive). The top row shows controls: unstained, PE Annexin V stained, and 7-AAD stained. The bottom row displays apoptosis progression among *Rp*_WT, *Rp_*Δ1266, and R_*Rp_*Δ1266-infected cells. (**B**) Quantitative comparison of apoptotic rates (percentage of apoptotic cells) across *Rp*_WT, *Rp_*Δ1266, and R_*Rp_*Δ1266-infected AAE2 cells. Data are presented as mean ± SD and analyzed using ANOVA, followed by the Bonferroni test to indicate significant differences. (**C**) Fold change in Caspase 3/7 activity relative to uninfected controls in *Rp*_WT, *Rp_*Δ1266, and R_*Rp_*Δ1266-infected AAE2 cells during later infection (120 hours post-infection), measured using a fluorescence plate reader. Data are presented as mean ± SD and analyzed using ANOVA, followed by the Bonferroni test to indicate significant differences.

To further investigate the role of *RPATATE_1266* in regulating apoptosis in tick cells, we restored the *RPATATE_1266* gene in *Rp_*Δ1266 (Fig. S4A). The restoration process involved electroporation of the *Rp_*Δ1266 mutant with the plasmid pRAM18dRGA carrying the *RPATATE_1266* gene, followed by selection on rifampicin and observation of positive green signals (GFPuv) under the fluorescent microscope. PCR and RT-PCR confirmed the successful restoration of the *RPATATE_1266* gene, resulting in the restored strain (R_*Rp_*Δ1266) (Fig. S4B through D). To evaluate the impact of *RPATATE_1266* restoration on apoptosis, we first used flow cytometry with PE Annexin V and 7-amino-actinomycin (7-AAD) staining to analyze early apoptosis (PE Annexin V positive, 7-AAD negative) and distinguish dead or necrotic cells (PE Annexin V and 7-AAD positive) in infected AAE2 cells. PE Annexin V binds to phosphatidylserine, a marker of early apoptosis (23), exposed on the outer leaflet of the plasma membrane, while 7-AAD penetrates and stains the DNA of cells with compromised membranes, identifying dead or necrotic cells. Early apoptotic cells are characterized as PE Annexin V positive and 7-AAD negative. Figure 5A displayed representative flow cytometry plots, with the Q3 quadrant corresponding to early apoptotic cells, and quantitative analysis from biological replicate experiments is presented in Fig. 5B. The results showed that the *Rp_*Δ1266-restored strain exhibited a higher early apoptotic rate compared to the *Rp_*Δ1266 mutant at 72 hours post-infection, although this increase was not statistically significant. Both the mutant and the restored strains demonstrated significantly lower early apoptotic rates compared to the *Rp_*WT strain, suggesting that while restoring *RPATATE_1266* slightly increases apoptosis relative to the mutant, it does not fully restore apoptosis to wild-type levels. To assess late-phase apoptosis, Caspase 3/7 activity, a specific marker for apoptotic processes unassociated with necrosis or other cell death pathways (24), was measured using a fluorescence plate reader at 120 hours post-infection. *Rp_*WT and R_*Rp_*Δ1266 exhibited significantly higher Caspase 3/7 activity compared to the *Rp_*Δ1266 mutant, indicating a strong late-phase apoptotic response in cells infected with wild-type *R. parkeri* and the restored strain. Notably, there were no significant differences in Caspase 3/7 activity between *Rp_*WT and R_*Rp_*Δ1266, demonstrating that *RPATATE_1266* restoration successfully rescues late-phase apoptosis (Fig. 5C). Taken together, these findings reveal that the *RPATATE_1266* gene plays a critical role in modulating apoptotic responses during infection, with its restoration partially rescuing early apoptosis and fully restoring late-phase apoptosis.

## DISCUSSION

Previously, we demonstrated that *R. parkeri* modulates mitochondrial-dependent apoptosis in tick cells, facilitating its intracellular survival and replication. However, the molecular mechanisms underlying this process remained unknown (8). Building on these findings, the current study identified the *RPATATE_1266* gene as a critical regulator of apoptosis in tick cells, advancing our knowledge of the key factors influencing *R. parkeri* infection dynamics. By employing a Himar1-based transposon mutagenesis system, we successfully disrupted the *RPATATE_1266* gene, revealing its essential role in the modulation of apoptosis and bacterial growth, and plaque formation. These results make great progress in studying the molecular interactions between *R. parkeri* and its tick vector, providing new insights into the pathogen’s intracellular survival strategies.

The generation of the *R. parkeri* RPATATE_1266 mutant (this study), along with the mutant RPATATE_0245::pLoxHimar (named 3A2) (14) and the mutant library (25), represents significant progress in the genetic study of *R. parkeri* using the Himar transposon system. However, the random insertion of the transposon into TA dinucleotide sites in the genome requires extensive lab work to screen for mutants. Additionally, this approach does not provide specific control over mutation sites or allow fine-tuning of gene expression, limiting its application in detailed genetic studies. Therefore, the development of advanced genetic tools is essential to further explore *Rickettsia* biology (26). Recently, the use of the Tet-On system and catalytically dead Cas9 (dCas9) in *R. parkeri* has marked a significant step in genetic manipulation for obligate intracellular pathogens (27). The application of dCas9 to knock down both antibiotic resistance genes and virulence factors like *sca2* in *R. parkeri* presents an exciting opportunity for more accurate and scalable genetic studies. Future research will employ the dCas9-based CRISPR interference (CRISPRi) to study *RPATATE_1266,* and applying this approach to other mutants generated through Himar1 mutagenesis could provide deeper insights into their role in *R. parkeri* survival, replication, and interactions with the vector.

Our evolutionary analysis of the *RPATATE_1266* gene revealed its conservation and functional importance. The presence of a single, highly conserved *RPATATE_1266* gene across all analyzed *Rickettsia* species, in contrast to the separate *ppx* and *gppa* genes in *E. coli* (28), suggests the evolutionary streamlining of the *Rickettsia* genome. Similar to *H. pylori* (19), this likely reflects adaptations to their obligate intracellular lifestyle, where metabolic and regulatory redundancy is minimized, and essential genes are conserved for survival within the restricted intracellular environment. The clustering of *Rickettsia* RPATATE_1266 proteins into a distinct phylogenetic clade further highlights the unique evolutionary pressures shaping the genus, separating it from other bacterial genera such as *Escherichia*, *Salmonella*, and *Helicobacter*. The high degree of conservation in functional domains, particularly those associated with the sugar kinase/actin/HSP70 superfamily, highlights the structural stability and multifunctional role of RPATATE_1266 in *Rickettsia* physiology. These findings suggest that RPATATE_1266 serves as a critical regulator of intracellular survival that may impact nucleotide metabolism, stress response, and adaptation to host-derived pressures. While the phylogenetic analysis provided valuable insights into the evolutionary conservation of RPATATE_1266, the functional differences between *Rickettsia* and other bacterial species remain unclear and require further experimental investigation.

Known for its role in (p)ppGpp metabolism and stringent response regulation, ppx/gppa participates in bacterial stress responses, metabolic processes, and intracellular survival (21). To explore its regulatory role, we analyzed predicted functional partner genes of *ppx/gppa* in six intracellular bacterial species (*Mycobacterium leprae*, *Neisseria meningitidis*, *Salmonella enterica*, *Shigella boydii*, *Francisella tularensis,* and *Yersinia pestis*) using the STRING database. Homologs of these functional partner genes were identified by searching against the *R. parkeri* genome (described in detail in Materials and Methods). The transcriptional shifts in these homologs suggest that the *Rp_*Δ1266 mutant exhibits an altered ability to regulate stress responses and metabolic pathways, likely due to changes in (p)ppGpp levels. Although this analysis primarily focused on gene expression changes associated with *RPATATE_1266*, it provides valuable new insights into the regulatory networks impacted by this gene in *R. parkeri*. STRING database predictions reveal overlapping functional partners, such as *relA*, *spoT*, *metG*, and *rpsD*, across various intracellular bacteria like *Neisseria meningitidis*, *Francisella tularensis*, and *Yersinia pestis*. These shared partners suggest conserved regulatory pathways among intracellular pathogens, reflecting their adaptation to host-induced stress. However, *R. parkeri* also shows unique differences in gene regulatory networks compared to other bacteria. For example, while *Shigella boydii* and *Salmonella enterica* associate *ppx/gppa* with genes such as *gppA*, *tehB*, and *purN*, these genes were not identified in *R. parkeri*. This divergence may reflect the distinct metabolic and regulatory requirements of *R. parkeri* in its tick vector and mammalian hosts (8, 29). The upregulation of relA, the primary (p)ppGpp synthase and a key regulator of the stringent response, in the *Rp_*Δ1266 mutant suggests that *R. parkeri* relies on RPATATE_1266 to balance (p)ppGpp metabolism. RelA synthesizes (p)ppGpp to initiate stringent response signaling, while GppA/Ppx homologs, such as RPATATE_1266, hydrolyze (p)ppGpp to regulate its levels. Disruption of RPATATE_1266 likely leads to accumulation of (p)ppGpp, resulting in altered transcriptional responses and stress adaptation mechanisms during intracellular survival. The transcriptional changes indicate that *RPATATE_1266* acts as a regulatory hub, modulating stress responses, metabolic pathways, and virulence-related genes in *R. parkeri*. However, we couldn’t measure (p)ppGpp levels or assess the impact of the mutation on substrate turnover due to the challenges of isolating sufficient quantities of bacterial proteins from obligate intracellular bacteria like *Rickettsia*, coupled with the lack of commercially available kits or reagents for such analyses.

Apoptosis is a highly regulated cellular process characterized by specific biomarkers such as gene expression changes, mitochondrial membrane potential (ΔΨm) changes, and DNA fragmentation. These biomarkers have been shown to work effectively in tick cell cultures in our previous study (8). Since *R. parkeri* utilizes mitochondrion-dependent apoptosis to facilitate its infection and replication, mitochondrial membrane potential (ΔΨm) changes provided particularly interesting insights in this study. The delayed mitochondrial dysfunction observed in *Rp_*Δ1266-infected cells validates the interplay between mitochondrial integrity and apoptosis regulation. Mitochondria are central to apoptotic signaling pathways, with ΔΨm changes often serving as an early indicator of apoptosis (30). Our findings suggest that *RPATATE_1266* helps maintain mitochondrial function during early infection (24 hours post-infection), likely contributing to bacterial replication by prolonging tick cell viability. However, the subsequent decline in ΔΨm at later infection (72 hours post-infection) in *Rp_*Δ1266-infected cells indicates that the inhibition of apoptosis is incomplete rather than entirely suppressed. This temporal regulation of apoptosis may reflect an adaptive strategy by *R. parkeri* to balance intracellular survival with host cell turnover in the tick vector. The role of *RPATATE_1266* in regulating apoptosis is further highlighted by its restoration in the mutant strain, which revealed differences between early- and late-phase responses. Restoration rescued *R. parkeri*’s ability to induce late-phase apoptosis, as Caspase 3/7 activity returned to levels similar to the wild-type *R. parkeri* (Fig. 5). While the complemented RPATATE_1266 strain successfully restored the late-phase apoptosis phenotype, it was not included in Fig. 2 and 3, which were designed to characterize the baseline phenotypic consequences of gene disruption in terms of bacterial growth and transcriptional regulation. Including the complemented strain in every phenotypic assay would have expanded the experimental scope considerably. Therefore, we prioritized its use in assays that directly evaluated apoptosis modulation, which is the central focus of this study. This targeted use allowed us to demonstrate the gene’s role in apoptosis regulation while maintaining a focused experimental framework. However, early-phase apoptosis (measured by Annexin V staining) showed no significant recovery in the *RPATATE_1266*-restored strain compared to the mutant. This difference may be due to our gene restoration approach, specifically the use of a shuttle plasmid to deliver the *RPATATE_1266* gene instead of reintegrating it into the bacterial genome. Unlike genomic integration, plasmid-based expression may be less stable or subject to different regulatory controls (12), potentially contributing to the differences in early- and late-phase apoptosis in tick cells.

The *R. parkeri RPATATE_1266* mutant exhibited reduced plaque formation (Fig. 2) along with delayed apoptosis in tick cells during early infection stages (Fig. 5). These findings highlight the role of apoptosis modulation in enhancing bacterial fitness within the tick vector by supporting intracellular survival and replication. Interestingly, this aligns with our previous study, which demonstrated that apoptosis activation in tick cells does not directly correlate with the pathogenicity of spotted fever group (SFG) *Rickettsia* species in humans (8). In the earlier study, we found that *R. helvetica*, *R. monacensis*, and *R. amblyommatis*—species with varying levels of human pathogenicity—showed different apoptotic responses in tick cells. For instance, *R. helvetica*, a human pathogen, did not significantly induce apoptosis in tick cells, whereas *R. amblyommatis*, a non-pathogenic symbiont, activated apoptosis in certain tick cell lines. This suggests that the modulation of apoptosis in tick cells is distinct from the mechanisms determining human pathogenicity, which likely involve additional factors beyond apoptosis activation. The delayed apoptotic response observed in *Rp_*Δ1266-infected tick cells further supports the hypothesis that apoptosis regulation in the vector is a localized adaptation to balance host cell viability and bacterial replication. While apoptosis modulation is a key strategy for survival and replication in the vector, it does not necessarily predict the pathogenicity of SFG *Rickettsia* in humans. These findings pave the way for further research to discover the mechanisms beyond vector adaptation and human pathogenicity in SFG *Rickettsia*.

In summary, this study identified the *RPATATE_1266* gene as a critical regulator of apoptosis, bacterial growth, and plaque formation in *R. parkeri*, providing new insights into the molecular interactions between rickettsial pathogens and their tick vectors. The findings emphasized the role of apoptosis modulation in bacterial fitness within the vector, highlighting its importance in intracellular survival and replication. Moving forward, further studies will utilize genetic tools, such as CRISPR-based systems, for targeted manipulation of *Rickettsia* genes and to elucidate the mechanistic links between *RPATATE_1266* activity, apoptotic regulation, and pathogen-host interactions. While this study focused on apoptosis due to its established relevance in tick—*R*. *parkeri* interactions and our prior observations, we acknowledge that RPATATE_1266 may influence additional host pathways. Broader transcriptomic analyses, such as RNA-seq, could reveal additional immune or metabolic processes impacted by this gene. Future studies using such approaches will help to further define the global regulatory role of RPATATE_1266 and its broader implications for pathogen-host interactions. Additionally, studies to explore the broader implications of apoptosis modulation across different vector-pathogen systems could discover conserved strategies employed by intracellular bacteria, paving the way for innovative approaches to control tick-borne diseases.

## MATERIALS AND METHODS

### Cell cultures

The tick cell lines AAE2 and ISE6 were cultured at 34°C in complete medium (L-15C300 supplemented with 5% fetal bovine serum [FBS], 5% tryptose phosphate broth, and 0.1% lipoprotein concentrate), as described previously (8). The Vero cell line was cultured at 37°C in complete RPMI1640 medium with 10% FBS.

### Growth and purification of *Rickettsia*

Wild-type *R. parkeri* (*Rp-*WT), the *Rp_*Δ1266 mutant strain, and the restored strain R*_ Rp_*Δ1266 were cultured in tick cells using complete medium with 10% FBS, 5% NaHCO₃, and 2.5% HEPES buffer. To purify *Rickettsia*, infected cells were transferred to 2.0 mL Eppendorf tubes containing 60–90 grit silicon carbide, vortexed at maximum speed for 30 seconds, and filtered through a Whatman 2.0 µm filter. The cell-free *Rickettsia* were collected by centrifugation at 13,000 × *g* for 5 minutes at 4°C and resuspended in complete medium. Viable *Rickettsia* were counted using a Petroff-Hausser chamber, following the manufacturer’s instructions.

### Generation of *R. parkeri RPATATE_1266* mutant

The *RPATATE_1266* mutant was generated using a Himar1-based transposon mutagenesis system. The pLoxHimar plasmid, designed for transposon mutagenesis in *Rickettsia* species, incorporates a well-characterized *Anaplasma marginale* promoter (*tr promoter*) to drive the co-expression of a fluorescent marker (mCherry) and a spectinomycin/streptomycin resistance gene (*aadA*) (31). This plasmid encodes both the transposase and transposon, configured to prevent transposase mobilization. Mismatched loxP sites flanking the mCherry and *aadA* genes facilitate potential transposon excision (14). To introduce the transposon into *R. parkeri*, bacteria were purified from ISE6 tick cell cultures and electroporated with 3 µg of the pLoxHimar plasmid following the standard transformation protocol (32). Transformed bacteria were cultured with ISE6 cells in 96-well plates with L15B300 medium supplemented with 10% FBS. Mutants expressing the fluorescent marker were selected with antibiotics for further analysis and confirmed by genomic DNA extraction, followed by PCR amplification and sequencing. Whole-genome sequencing was performed to validate the location of the transposon insertion.

### Phylogenetic analysis of RPATATE_1266

To investigate the evolutionary relationship of the RPATATE_1266 protein in *R. parkeri*, amino acid sequences from 38 bacterial species (listed in Table S1) were retrieved from NCBI and Ensembl Bacteria (https://bacteria.ensembl.org/index.html). Phylogenetic analysis was performed using the maximum likelihood method in PhyML-3.1 software (33) with 1000 bootstrap replications. Multiple sequence alignment of Ppx/Gppa homolog protein sequences from selected bacterial species, including *Rickettsia typhi*, *R. prowazekii*, *R. parkeri*, *R. rickettsii*, *Escherichia coli*, and *Helicobacter pylori*, was conducted using ClustalW v2.0 (34, 35) with default parameters.

### Giemsa staining

Infected AAE2 cells (*Rp_*WT and *Rp_*Δ1266) were resuspended and centrifuged onto glass slides using a cytocentrifuge at 1,200 rpm for 3 minutes, then air-dried. The slides were fixed in methanol for 5 minutes, stained with Giemsa solution (Sigma) for 30 minutes at 37°C, and rinsed gently with water. After air-drying, the slides were visualized under oil immersion at 100× magnification, with *Rickettsia* appearing purple against a pink background, for infection rate assessment.

### Rickettsia DNA extraction and qPCR

Genomic DNA was extracted from cell-free *Rickettsia* using the DNeasy Blood & Tissue Kit (Qiagen), following the manufacturer’s protocol for Gram-negative bacteria. The quantity and quality of the extracted DNA were assessed using a DS-11 Series Spectrophotometer/Fluorometer (DeNovix). The copy number of the single-copy *Rickettsia* citrate synthase (*gltA*) gene was quantified by qPCR on a Bio-Rad CFX96 Real-Time System (Bio-Rad) using iTaq Universal SYBR Green Supermix (Bio-Rad). Primers used for amplification are listed in Table S2.

### Inverse PCR

The genomic sequence of *R. parkeri* (Tate’s Hell) was retrieved from the NCBI database (GCA_000965145.1) and analyzed using SnapGene to identify suitable restriction enzymes (AseI and SspI) that would fragment the DNA into 3,000–5,000 base pair segments without cutting within the insertion sequence. Genomic DNA was digested with AseI and SspI under recommended conditions, and the resulting fragments were purified using the PCR Clean-Up Kit (Zymo Research) to remove residual enzymes and impurities. Purified DNA fragments were ligated using DNA ligase under optimized conditions for cohesive-end ligation. Insertion-specific primers were designed to read out from the transposon insertion site (36) to amplify the region surrounding the transposon. PCR products were analyzed by agarose gel electrophoresis to confirm the amplified fragments and verify the insertion site.

### Rickettsia RNA isolation and qRT-PCR

Total RNA was extracted from cell-free *Rickettsia* using TRI Reagent (Sigma), following the manufacturer’s instructions. RNA quantity and quality were evaluated using a DS-11 Series Spectrophotometer/Fluorometer (DeNovix). Complementary DNA was synthesized using the SYBR PrimeScript RT-PCR Kit II (Takara). Gene expression levels regulated by *RPATATE_1266* were quantified using qPCR on a Bio-Rad CFX96 Real-Time System with iTaq Universal SYBR Green Supermix (Bio-Rad). Primers used for amplification are listed in Table S2.

### Regulatory role of RPATATE_1266 in gene expression (GRNs)

The STRING database (22) was used to predict gene regulatory networks (GRNs) associated with *RPATATE_1266*. Functional partner genes were identified for six intracellular bacterial species (*Mycobacterium leprae, Neisseria meningitidis, Salmonella enterica, Shigella boydii, Francisella tularensis*, and *Yersinia pestis*) (listed in Table S3). Homologues of these functional partner genes were then identified by performing BLAST searches against the *R. parkeri* genome. Gene expression changes associated with RPATATE_1266 were analyzed by qRT-PCR as described above. Relative expression levels were calculated using the ∆∆Ct method, normalized to the 16S rRNA gene. Primers used for amplification were designed based on the *R. parkeri* Tate’s Hell genome and are listed in Table S2.

### Plaque assay

Vero cells were seeded in 6-well plates and incubated until reaching ~95% confluency. Bacterial suspensions of *Rp*_WT and *Rp_*Δ1266 were prepared from cell-free extracts and quantified by using a Petroff-Hausser counting chamber. Bacterial concentrations were adjusted to achieve equivalent numbers based on counts to ensure similar input loads across strains. Serial 10-fold dilutions (10⁻¹ to 10⁻⁷) of *Rickettsia* (*Rp*_WT and *Rp_*Δ1266) were prepared, and 100 µL of each dilution was added to the wells. After a 2 hour incubation at 37°C to allow bacterial attachment, the wells were washed with warm DMEM to remove unbound bacteria and overlaid with a 1% agarose-DMEM mixture to restrict bacterial spread (14). Plates were then incubated at 37°C for 4 days. Plaques (~1 mm clear areas) were counted under an inverted microscope at the 10⁻⁵ dilution in duplicate wells. All treatments were replicated three times.

### Restoration of *RPATATE_1266* in *R. parkeri*

The *RPATATE_1266* gene was restored in *Rp_*Δ1266 through electroporation, as previously described (32). The mutant strain was transformed with the plasmid pRAM18dRGA carrying the *RPATATE_1266* gene, based on the method successfully applied in *R. parkeri* (37). After electroporation, bacteria were recovered in L15B300 medium supplemented with 10% FBS and co-cultured with ISE6 tick cells in 96-well plates. Transformants were selected by adding rifampicin to the medium and screening for the expression of the fluorescent marker (GFPuv). Positive transformants (with green signals under a fluorescence microscope) were confirmed through genomic DNA extraction, followed by PCR amplification and sequencing of the *RPATATE_1266* gene. To ensure the purity of the mutant population and avoid potential confounding from residual WT *RPATATE_1266*, plaque purification with rifampicin selection was performed to isolate a single clone. Transcription of the restored *RPATATE_1266* gene was verified using RT-PCR with primers listed in Table S2.

### Mitochondrial membrane depolarization detection

Mitochondrial membrane potential (∆Ψm) loss was evaluated using the JC-1 dye (5,5′,6,6′-tetrachloro-1,1′,3,3′-tetraethylbenzimidazolocarbocyanine iodide; Immunochemistry), following the manufacturer’s protocol. AAE2 cells were infected with cell-free *R. parkeri* strains (*Rp*_WT and *Rp_*Δ1266) at a multiplicity of infection of 1 for 24 or 72 hours. Uninfected AAE2 cells served as a negative control, while uninfected cells treated with CCCP were included as a positive control for mitochondrial depolarization. After incubation, samples were stained with JC-1 and analyzed for a red-to-green fluorescence shift (red ~590 nm; green ~529 nm) using a Biotek M3 fluorescence plate reader. All treatments were replicated three times.

### TUNEL assay

Apoptotic cell death was detected using the *In Situ* Cell Death Detection Kit (Roche) following the manufacturer’s protocol. Cells were fixed on slides with 4% paraformaldehyde, permeabilized with 0.1% Tween 20 in PBS, and incubated with TUNEL reagents (TdT enzyme and dUTP mix, 1:10) at 37°C for 1 hour. After staining, slides were mounted with DAPI-containing Fluoroshield (Vector Laboratories) and imaged using an Olympus BX61 DSU confocal microscope with a 60× objective (imaging settings as described in reference 8). All treatments were replicated three times.

### Flow cytometry

Flow cytometry was used to evaluate apoptosis in *Rickettsia*-infected AAE2 tick cells with the PE Annexin V/7-AAD Apoptosis Detection Kit (BD Biosciences). Cells were separately infected with *Rp*_WT, *Rp_*Δ1266, and R*_ Rp_*Δ1266, with uninfected cells as a negative control. After 72 hours of infection, cells were harvested, washed twice with cold 1× PBS, and resuspended in 300 µL of 1× Annexin V Binding Buffer at a density of 1 × 10⁶ cells/mL. For staining, 5 µL each of Annexin V-PE and 7-AAD was added to 100 µL of the cell suspension. Samples were incubated in the dark for 15 minutes and then diluted with 400 µL of Binding Buffer before analysis. Controls included unstained cells, single-stained cells for compensation settings, and a positive apoptosis control treated with 1 µM paclitaxel for 1 hour. Data acquisition was performed on a BD LSRII flow cytometer, analyzing 10,000 events per sample. Data were processed with BD FACSDiva. Cells were classified as viable, early apoptotic, late apoptotic, or necrotic based on Annexin V and 7-AAD staining. Signal compensation and gating were optimized using controls, and apoptotic populations were quantified for statistical comparison. The flow cytometry results were analyzed using FlowJo v10.8 Software (BD Life Sciences). All treatments were replicated three times.

### Caspase 3/7 enzyme activity assay

Caspase 3/7 enzyme activity was measured using the Magic Red Caspase 3/7 Assay Kit (Immunochemistry) according to the manufacturer’s instructions. AAE2 cells were separately infected with *Rp*_WT, *Rp_*Δ1266, and R*_ Rp_*Δ1266, while uninfected cells served as controls. After 120 hours of infection, cells were incubated with the Magic Red substrate, then fluorescence intensity (caspase activity) was measured using a Biotek M3 fluorescence plate reader with excitation at ~550 nm and emission at ~590 nm. All treatments were replicated three times.

### Statistical analysis

We applied one-way analysis of variance (ANOVA) for analysis of all data of gene expression, percentages of apoptotic cells, the quantities of *rickettsia* DNA, infection rate, plaque assay, the ratio of red/green fluorescence, and the Caspase 3/7 enzyme activity. This was followed by Bonferroni analysis when there were ≥3 treatments. The Student’s two-tailed *t*-test was applied for the analysis when there were only two treatments. Differences were judged significant when *P* < 0.05. All statistical analyses were performed using GraphPad Prism software.

## SUPPLEMENTAL MATERIAL

Supplemental figure legends spectrum.00151-25-s0001.docx Legends for Fig. S1 to S4.

Figure S1 spectrum.00151-25-s0002.pdf Phylogenetic analysis of Ppx/Gppa across 38 bacterial species.

Figure S2 spectrum.00151-25-s0003.pdf Multiple sequence alignment of Ppx/Gppa from selected bacterial species.

Figure S3 spectrum.00151-25-s0004.pdf Activation of apoptosis by intergenic mutant *R. parkeri* infection.

Figure S4 spectrum.00151-25-s0005.pdf Restoration of RPATATE_1266 gene in mutant *R. parkeri*.

Table S1 spectrum.00151-25-s0006.docx List of bacterial species and associated protein accession numbers used for phylogenetic analysis of RPATATE_1266 homologs.

Table S2 spectrum.00151-25-s0007.docx Primers related to Methods.

Table S3 spectrum.00151-25-s0008.docx Predicted functional partner genes associated with ppx/gppa in six intracellular bacterial species and selected homologues in *R. parkeri*, related to Methods.

ASM does not own the copyrights to Supplemental Material that may be linked to, or accessed through, an article. The authors have granted ASM a non-exclusive, world-wide license to publish the Supplemental Material files. Please contact the corresponding author directly for reuse.
