# Supplementary material for: Rickettsia parkeri hypothetical protein RPATATE_1266, a homolog of exopolyphosphatase/guanosine pentaphosphate phosphohydrolase, regulates tick cell apoptosis
Source: Microbiol Spectr. 2025 Jul 7;13(8):e00151-25. doi: 10.1128/spectrum.00151-25 (PMC12323366; doi:10.1128/spectrum.00151-25)
Supplement: Supplemental figure legends — Legends for Fig. S1 to S4. [file spectrum.00151-25-s0001.docx]

**SUPPLEMENTAL MATERIAL**

**Supplementary Table 1.** List of bacterial species and associated **protein accession numbers** used for phylogenetic analysis of RPATATE_1266 homologs. Amino acid sequences were retrieved from NCBI and Ensembl Bacteria based on homology to Ppx/Gppa family proteins. These sequences were used for multiple sequence alignment and maximum likelihood phylogenetic tree construction (as described in the Methods section).

**Supplementary Table 2.** Primers, related to Methods.

**Supplementary Table 3.** Predicted functional partner genes associated with *ppx/gppa* in six intracellular bacterial species and selected homologues in *R. parkeri*, related to Methods.

**Supplementary Figures**

**Fig. S1. Phylogenetic analysis of Ppx/Gppa across 38 bacterial species.**

An unrooted phylogenetic tree was constructed to analyze Ppx/Gppa sequences from 38 bacterial species, representing genera such as *Campylobacter*, *Helicobacter*, *Escherichia*, *Salmonella*, and various *Rickettsia* species. Phylogenetic trees were generated using the maximum likelihood method with 1000 bootstrap replications in PhyML-3.1 software.

**Fig. S2. Multiple sequence alignment of RPATATE_1266 (Ppx/Gppa homologs) from selected bacterial species.**Amino acid sequences were aligned using ClustalW to compare RPATATE_1266 homologs from various Rickettsia species (*R. typhi*, *R. prowazekii*, *R. parkeri*, and *R. rickettsii*), *E. coli*, and *H. pylori*. Conserved residues are shaded, with darker shading indicating higher conservation. Red underlines indicate conserved domains predicted to belong to the sugar kinase/actin/HSP70 superfamily. This domain is commonly associated with enzymes involved in nucleotide metabolism and stress response. Black-highlighted residues within these domains may contribute to the structural integrity and potential enzymatic activity, including interaction with nucleotide-derived molecules such as (p)ppGpp. This alignment supports the domain's evolutionary conservation and potential functional relevance across intracellular bacterial species.

**Fig. S3. Activation of apoptosis by intergenic mutant *R. parkeri* infection.**

**(A):** Identification of the pLoxHimar transposon insertion site in the genome of the *R. parkeri* intergenic mutant by sequencing. The insertion site is located between two genes: the p-type conjugative transfer protein *VirB9* and *NADH-ubiquinone/plastoquinone*. **(B):** TUNEL staining (green) of AAE2 cells infected with the *R. parkeri* intergenic mutant, indicating apoptotic cells. DAPI staining (blue) marks the nuclei. Scale bar: 20 µm. **(C):** Quantification of apoptotic cells (TUNEL-positive/DAPI-positive cells) across different fields. Data are presented as mean ± SD and analyzed using ANOVA followed by the Bonferroni test. Different letters above the columns indicate significant differences.

**Fig. S4. Restoration of *RPATATE_1266* gene in mutant *R. parkeri***.

**(A)** Schematic overview of the complementation strategy. The RPATATE_1266 mutant strain of *R. parkeri* (*Rp*_*Δ1266*) was electroporated with plasmid pRAM18dRGA carrying the RPATATE_1266 gene (homologous to ppx/gppa). Transformants were selected on rifampicin and screened using fluorescence microscopy, PCR, and sequencing. **(B)** PCR validation of RPATATE_1266 gene presence in wild-type (WT), mutant, and complemented strains using gene-specific primers. Lane details: 1 – 1 kb DNA ladder; 2 – PCR product from WT genomic DNA (gDNA); 3 – PCR product from mutant gDNA; 4 – PCR product from complemented mutant gDNA. **(C)** PCR confirmation of shuttle plasmid presence using plasmid-specific primers. Lane details:1 – 1 kb DNA ladder; 2 – PCR product from WT gDNA; 3 – PCR product from mutant gDNA; 4 – PCR product from complemented mutant gDNA. **(D)** RT-PCR verification of RPATATE_1266 expression in WT and mutant strains. Lane details: 1 – 1 kb DNA ladder; 2 – RT-PCR product from WT RNA; 3 – RT-PCR product from mutant RNA; 4- RT-PCR product from complemented mutant RNA.
