# Supplementary figures and images for: Rickettsia parkeri hypothetical protein RPATATE_1266, a homolog of exopolyphosphatase/guanosine pentaphosphate phosphohydrolase, regulates tick cell apoptosis
Source: Microbiol Spectr. 2025 Jul 7;13(8):e00151-25. doi: 10.1128/spectrum.00151-25 (PMC12323366; doi:10.1128/spectrum.00151-25)

**Fig.S1**

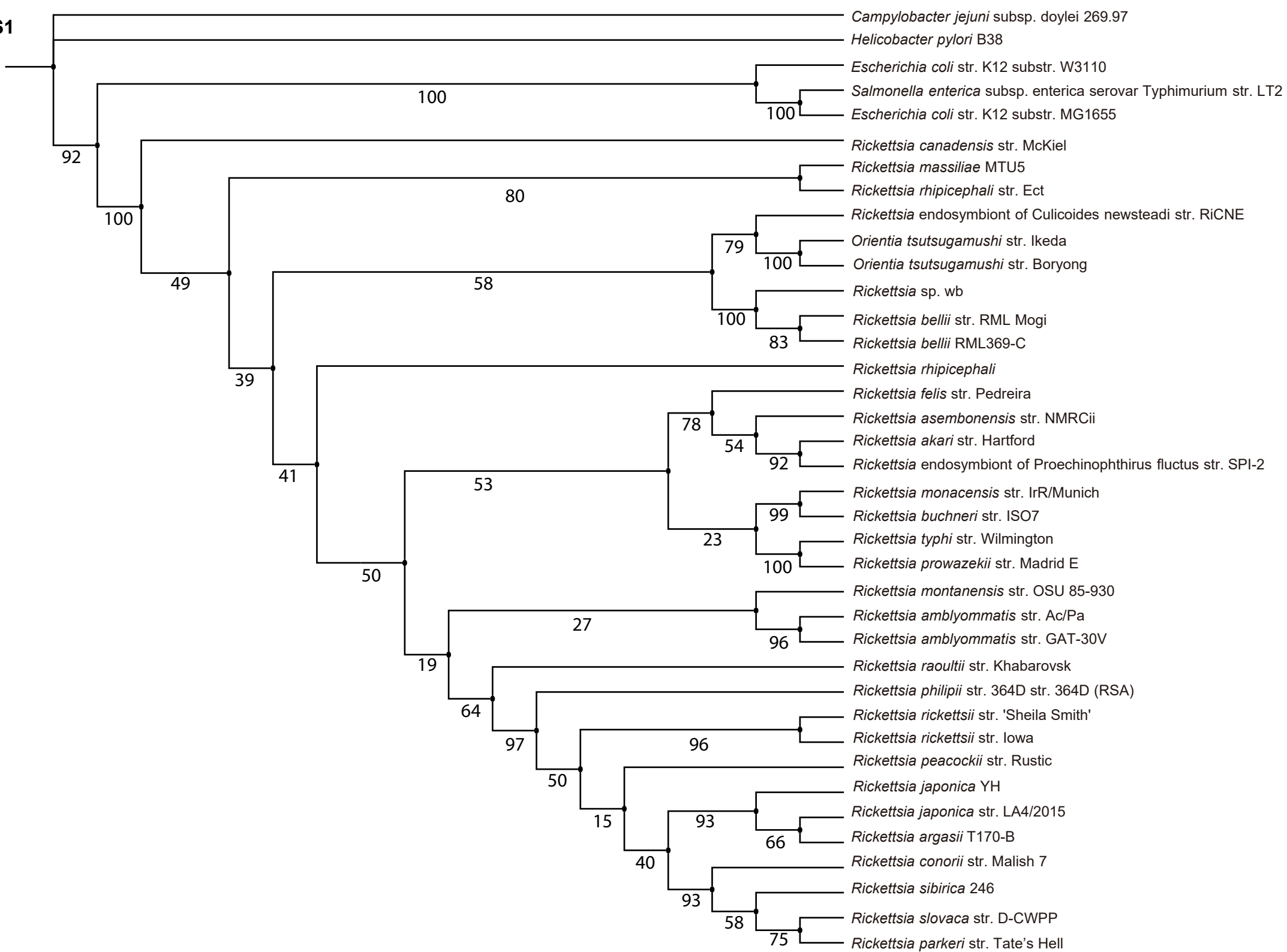

Supplement: Figure S1 — Phylogenetic analysis of Ppx/Gppa across 38 bacterial species. [file spectrum.00151-25-s0002.pdf]

**Fig. S3**

**A**

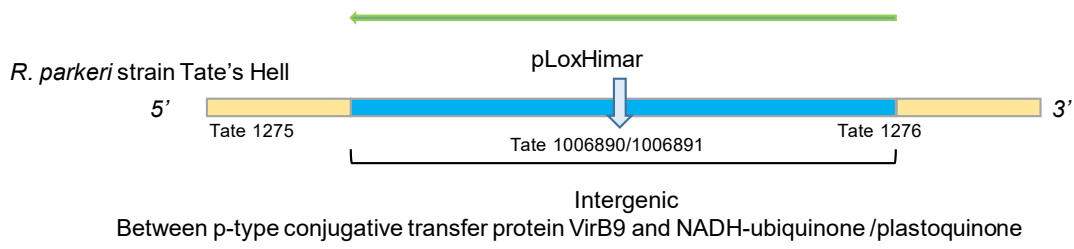

**B**

intergenic mutant *R. parkeri*

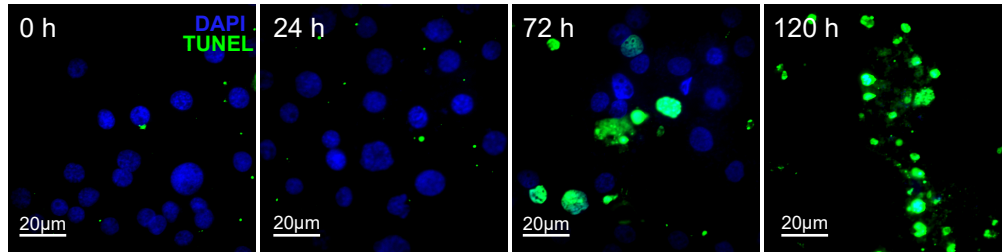

**C**

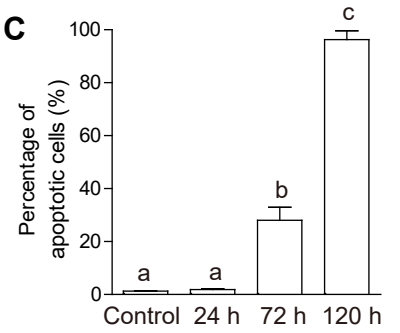

Supplement: Figure S3 — Activation of apoptosis by intergenic mutant R. parkeri infection. [file spectrum.00151-25-s0004.pdf]

Fig.S4

A

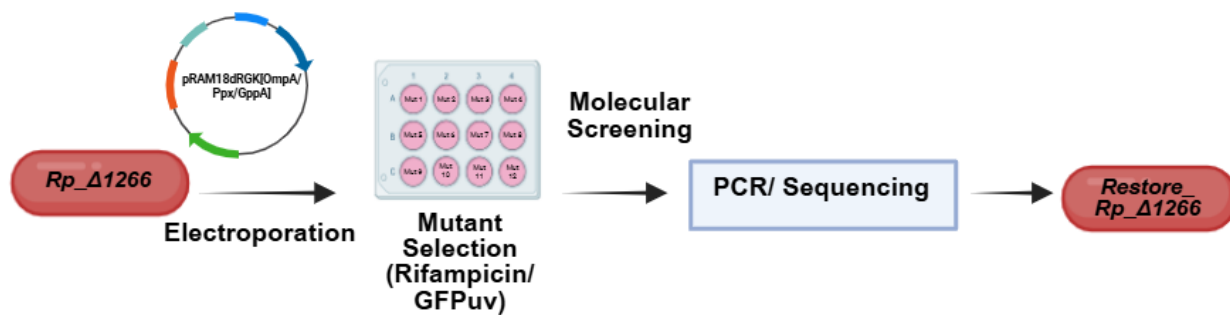

B

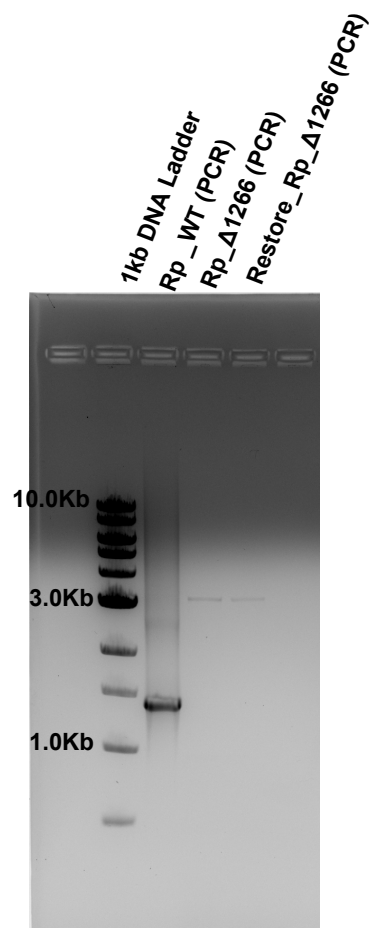

C

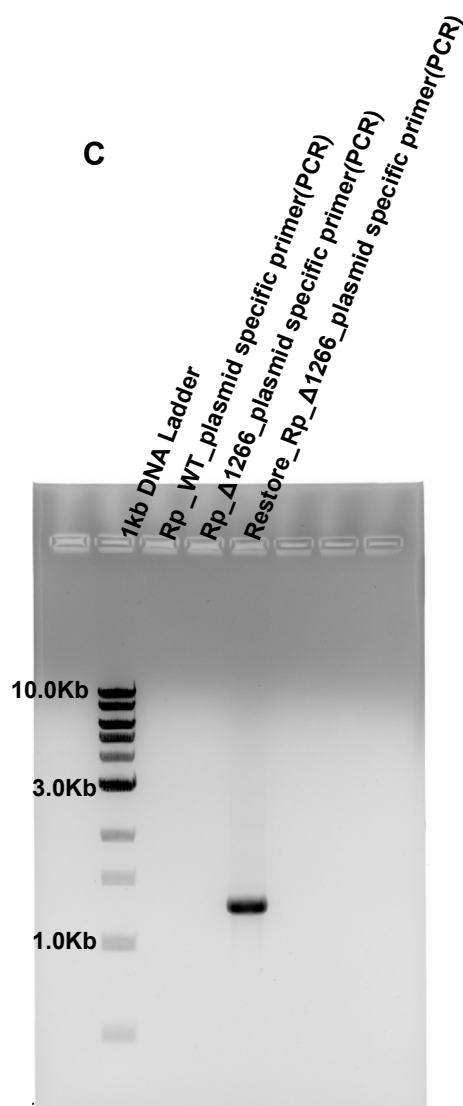

D

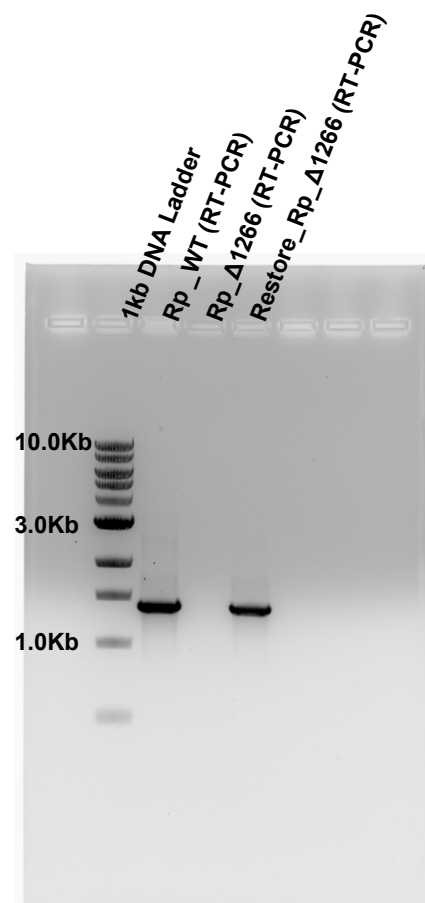

Supplement: Figure S4 — Restoration of RPATATE_1266 gene in mutant R. parkeri. [file spectrum.00151-25-s0005.pdf]
