# Supplementary material for: Rickettsia parkeri hypothetical protein RPATATE_1266, a homolog of exopolyphosphatase/guanosine pentaphosphate phosphohydrolase, regulates tick cell apoptosis
Source: Microbiol Spectr. 2025 Jul 7;13(8):e00151-25. doi: 10.1128/spectrum.00151-25 (PMC12323366; doi:10.1128/spectrum.00151-25)
Supplement: Figure S2 — Multiple sequence alignment of Ppx/Gppa from selected bacterial species. [file spectrum.00151-25-s0003.pdf]

Fig. S2

```
                     *       20         *        40         *        60         *        80         *       100         *       120         *
Rt_PPX/GppA  : ----------MRSAIIDIGSNALRAVVYESD-ELGAPEIFNYKFRNYLTN-LLNLDN---LDVKHQTYLSIQYLIHVFTKLSVTNIKCVATAILRGHPKADEFKTIIKKRFNIDIEIISGEREAYLTAAGLI : 117
Rpr_PPX/GppA : ----------MRSAIIDIGSNALRAVVYESD-ELGAPEIFNYKFRNYLTN-LLNLDN---LDVKHQTYLSIQYLIHVFTKLSVTNIRCVATAILRGHPKADEFKTIIKKRFNIDIEIISGEREAYLTAAGLI : 117
Rpa_PPX/GppA : ----------MRSAIIDIGSNALRAVVYESD-ELGAPEIFNYKFRNYLTN-LLNLDN---LDVKHQTYLSIQYLIHIFTKLSVTNIRCVATAILRGHPKADEFKAIIKKRFNIDIEIISGEREAYLTAAGLI : 117
Rr_PPX/GppA  : -------MSKMRSAIIDIGSNALRAVVYESD-ELGAPEIFNYKFRNYLTN-LLNLDN---LDVKHQTYLSIQYLIHIFTKLSVTNIRCVATAILRGHPKADEFKAIIKKRFNIDIEIISGEREAYLTAAGLI : 120
E.coli_PPX   : MPIHDKSPRPQEFAAVDLGSNSFHMVIARVV-DGAMQIIGRLKQRVHLAD-GLGPDNMLSEEAMTRGLNCLSLFAERLQGFSPASVCIVGTHTLRQALNATDFLKRAEKVIPYPIEIISGNEEARLIFMGVE : 130
E.coli_GppA  : -----MGSTSSLYAAIDLGSNSFHMLVVREV-AGSIQTLTRIKRKVRLAA-GLNSENALSNEAMERGWQCLRLFAERLQDIPPSQIRVVATATLRLAVNAGDFIAKAQEILGCPVQVISGEEEARLIYQGVA : 125
Hp_PPX/GppA  : --------MAKITTVIDIGSNSVRLAIFKKTSQFGFYLLFETKSKVRISEGCYAFNGILQEIPMQRAVKALSEFKEIALKYKSKKILCVATSAVRDAPNRLEFVARVKKACGLQIKIIDGQKEALYGGIACA : 124

                   140          *       160         *       180         *       200         *       220         *       240         *       260
Rt_PPX/GppA  : SGISDAFG-IVADLGGGSLEIAHICNKKVGKIKSLPLGTKIITNSNVGDVGLITKMLEEEFGVAHYP-----NLYLIGGALRLMSRIYMESINYPLKNLHN--FEINRVEFELYLEKISQIDKLKLSY---- : 237
Rpr_PPX/GppA : SGISDAFG-IVADLGGGSLEIAHIYNKKVGKIKSLPLGTKIIANSNFSDVGLITKMLEEEFGVAHYP-----NLYLIGGALRLMSRIYMESINYPLKNLHN--FEINRVEFELYLEKISQIDKLKLSY---- : 237
Rpa_PPX/GppA : SGISDAFG-IVADLGGGSLEIAQIGNKKVGKIKSLPLGTKIIVSSNFGDVGLITKMLEEEFGAAHYP-----NLYLIGGALRLMSRIYMESINYPLKNLHN--FEINRVEFELYLEKISQIDKLKLSY---- : 237
Rr_PPX/GppA  : SGISDAFG-IVADLGGGSLEIAQIGNKKVGKIKSLPLGTKIIVSSNFGDVGLITKMLEEEFGAAHYP-----NLYLIGGALRLMSRIYMESINYPLKNLHN--FEINRVEFELYLEKISQIDKLKLSY---- : 240
E.coli_PPX   : HTQPEKGRKLVIDIGGGSTELVIGENFEPILVESRRMGCVSFAQLYFPGG-VINKENFQRARMAAAQ-----KLETLTWQFRIQGWNVAMGASGTIKAAHEVLMEMGEKDGIITPERLEKLVKEVLRHRNFA : 256
E.coli_GppA  : HTTGGADQRLVVDIGGASTELVTGTGAQTTSLFSLSMGCVTWLERYFADR-NLGQENFDAAEKAARE-----VLRPVADELRYHGWKVCVGASGTVQALQEIMMAQG-MDERITLEKLQQLKQRAIHCGRLE : 250
Hp_PPX/GppA  : NLLHKNSG-ITIDIGGGSTECALIEKGKIKDLISLDVGTIRIKEMFLDKDLEVKLAKAFIQKEVSKLPFKHKNAFGVGGTIRALSKILMKRFDYPIDSLHG--YEIDAYKNLAFIEKIVMLKEDQLRLLG-- : 251

                  *       280         *       300         *       320         *       340         *       360         *       380         *
Rt_PPX/GppA  : --------YEQKAINYNAVLVIKAMLKVFSPEKIIISNYGLKEGVRFDSLP--YHETEK---DIIYERVKRLVNFDKNICKIEKYIEALQYLLIN------SDATTLIIIELAIMLAQYNKNIDKTLRANFV : 350
Rpr_PPX/GppA : --------YEQKAINYNAVLVIKAMIKVFSPEKIIISNYGLKEGVRFDSLP--YHETEK---DIIYERVKRLVNFDRNICKIEKYIEALQYLLIN------SDATTLIIIELAIMLAQYNKNIDKTLRANFV : 350
Rpa_PPX/GppA : --------YEQKVINYNAVLVIKAMIKVFSPEKIIISNYGLKEGVRFDSLP--SHETEK---DIIYERVKRLVKFDRNICKIEKYIEAVQYLLIN------ADATTLIVIELAIMLAQYNKNIDKTLRANFV : 350
Rr_PPX/GppA  : --------YEQKVINYNAVLVIKAMIKVFSPEKIIISNYGLKEGVRFDSLP--SHETEK---DIIYERVKRLVKFDRNICKIEKYIEAVQYLLIN------ADATTLIIIELAIMLAQYNKNIDKTLRANFV : 353
E.coli_PPX   : SLSLPGLSEERKTVFVPGLAILCGVFDALAIRELRLSDGALREGVLYEMEGRFRHQDVR---SRTASSLANQYHIDSEQARRVLDTTMQMYEQWREQQPKLAHPQLEALLRWAAMLHEVGLNINHSGLHRHS : 385
E.coli_GppA  : ELEIDGLTLERALVFPSGLAILIAIFTELNIQCMTLAGGALREGLVYGMLHLAVEQDIR---SRTLRNIQRRFMIDIDQAQRVAKVAANFFDQVENEWHLEAISR--DLLISACQLHEIGLSVDFKQAPQHA : 377
Hp_PPX/GppA  : ------VNEERLDSIRSGALIILSVVLEHLKTSLMITSGVGVREGVFLSDILRHHYHKFPPNINPSLISLKDRFLPHEKHSQKVKKECVKLFEALSP--LHKIDEKYLFHLKIAGELASMGKILSVYLAHKHS : 375

                 400          *       420         *       440         *       460         *       480         *       500         *       520
Rt_PPX/GppA  : SEFILSSDIP-FSQRQRLMLGIALTVTYTAKTDMQINKIAKKMISKSDYYNSHIIGYYIKIAREIDGPEFQEPSFSIKLKDDKFLQINASN------ILPKQVFDKVCERLKDISSARKNISYNFSD---- : 470
Rpr_PPX/GppA : SEFILSSDIP-FSHRQRLMLGIALTVTYTAKTDMQINKIAKKMISKSDYYNSHIIGYYIKIAREIDGPEFQEPSFSIKLKDDKFLQINASS------ILPKQVFDKVCERLKDISSARKNISYNFSD---- : 470
Rpa_PPX/GppA : SEFILSSDIP-FSHRQRLMLGIALTVTYTAKTDMQINKIAKKMISKSDYYNSHIIGYYIKIAREIDGPEFQEPSFSIKLKDDKFLQINASN------ILPKQVFEKVCERLKDISSARKNIRYNFSD---- : 470
Rr_PPX/GppA  : SEFILSSDIP-FSHRQRLMLGIALTVTYTAKTDMHINKIAKKMISKSDYYNSHIIGYYIKIAREIDGPEFQEPSFSIKLKDDKFLQINASN------ILPKQVFEKVCERLKDISSARKNIRYNFSD---- : 473
E.coli_PPX   : AYILQNSDLPGFNQEQQLMM---ATLVRYHRKAIKLDDLPRFTLFKKKQFLPLIQLLRLGVLLNNQRQATTTPPTLTLITDDSHWTLRFPHDWFSQNALVLLDLEKEQEYWEGVAGWRLKIEEESTPEIAA : 513
E.coli_GppA  : AYLVRNLDLPGFTPAQKKLL---ATLLNQTNPVDLSSLHQQNAVPPRVAEQLCRLLRLAIIFASRRRDDLVP-EMTLQANHELLTLTLPQGWLTQHPLGKEIIAQESQ-WQSYVHWPLEVH--------- : 494
Hp_PPX/GppA  : AYFILNALSYGFSHQDRAIIC--LLAQFSHKKIPKDNAIAHMSAMMP----SLLTLQWLSFILSLAENLCLTDSHHLKYTLEKNKLVIHSN---------DALYLAKEMLPKLVKPIPLTIEFA------- : 484
```
