# Supplementary material for: Rickettsia parkeri hypothetical protein RPATATE_1266, a homolog of exopolyphosphatase/guanosine pentaphosphate phosphohydrolase, regulates tick cell apoptosis
Source: Microbiol Spectr. 2025 Jul 7;13(8):e00151-25. doi: 10.1128/spectrum.00151-25 (PMC12323366; doi:10.1128/spectrum.00151-25)
Supplement: Table S1 — List of bacterial species and associated protein accession numbers used for phylogenetic analysis of RPATATE_1266 homologs. [file spectrum.00151-25-s0006.docx]

**Table S1**: List of bacterial species and associated **protein accession numbers** used for phylogenetic analysis of RPATATE_1266 homologs. Amino acid sequences were retrieved from NCBI and Ensembl Bacteria based on homology to Ppx/Gppa family proteins. These sequences were used for multiple sequence alignment and maximum likelihood phylogenetic tree construction (as described in the Methods section).

| **Species of** **Bacteria** | **Protein Accession Number** |
| --- | --- |
| *Campylobacter jejuni* subsp. doylei 269.97 (GCA_000017485) | ABS44229 |
| *Helicobacter pylori* B38 (GCA_000091345) | CAX28828 |
| *Escherichia coli* str. K-12 substr. W3110 (GCA_000335215) | BAA16390 |
| *Salmonella enterica* subsp. enterica serovar Typhimurium str. LT2 (GCA_000006945) | AAL22763 |
| *Escherichia coli* str. K-12 substr. MG1655 (GCA_000005845) | AAT48210 |
| *Rickettsia canadensis* str. McKiel (GCA_000014345) | ABV73756 |
| *Rickettsia massiliae* MTU5 (GCA_000016625) | ABV84645 |
| *Rickettsia rhipicephali* str. Ect (GCA_000964905) | KJV79422 |
| *Rickettsia* *endosymbiont of Culicoides newsteadi* str. RiCNE (GCA_002259525) | OZG32457 |
| *Orientia tsutsugamushi* str. Ikeda (GCA_000010205) | BAG39705 |
| *Orientia tsutsugamushi* str. Boryong (GCA_000063545) | CAM79531 |
| *Rickettsia sp. wb* (GCA_001707925) | ODA38041 |
| *Rickettsia bellii* str. RML Mogi (GCA_000965045) | KJV91513 |
| *Rickettsia bellii* RML369-C (GCA_000012385) | ABE05100 |
| *Rickettsia rhipicephali* (GCA_001442475) | ALN41515 |
| *Rickettsia felis* str. Pedreira (GCA_000964665) | KJV58013 |
| *Rickettsia asembonensis* str. NMRCii (GCA_000828125) | KIJ88382 |
| *Rickettsia akari* str. Hartford (GCA_000018205) | ABV74727 |
| *Rickettsia* *endosymbiont of Proechinophthirus fluctus* str. SPI-2 (GCA_001602635) | KYP98562 |
| *Rickettsia monacensis* str. IrR/Munich (GCA_000499665) | CEO17943 |
| *Rickettsia buchneri* str. ISO7 (GCA_000696365) | KDO02695 |
| *Rickettsia typhi* str. Wilmington (GCA_000008045) | AAU03765 |
| *Rickettsia prowazekii* str. Madrid E (GCA_000195735) | CAA14755 |
| *Rickettsia montanensis* str. OSU 85-930 str. OSU 85-930 (RMO) (GCA_000284175) | AFC74037 |
| *Rickettsia amblyommatis* str. Ac/Pa (GCA_000964675) | KJV61838 |
| *Rickettsia amblyommatis* str. GAT-30V str. GAT-30V (RAM) (GCA_000284055) | AFC69517 |
| *Rickettsia raoultii* str. Khabarovsk (GCA_000940955) | AJQ51668 |
| *Rickettsia philipii* str. 364D str. 364D (RSA) (GCA_000283995) | AFB26025 |
| *Rickettsia rickettsii* str. 'Sheila Smith' (GCA_000018225) | ABV76004 |
| *Rickettsia rickettsii* str. Iowa (GCA_000017445) | ABY72353 |
| *Rickettsia peacockii* str. Rustic (GCA_000021525) | ACR47545 |
| *Rickettsia japonica* YH (GCA_000283595) | BAK96530 |
| *Rickettsia japonica* str. LA4/2015 (GCA_003454715) | AXU07120 |
| *Rickettsia argasii* T170-B (GCA_000965185)  *Rickettsia conorii* str. Malish 7 (GCA_000007025)  *Rickettsia sibirica* 246 (GCA_000166935)  *Rickettsia slovaca* str. D-CWPP str. D-CWPP (RSB) (GCA_000252365)  *Rickettsia parkeri* str. Tate's Hell (GCA_000965145.1) | KJW05508  AAL02930  EAA25560  AFD19411  WP_012719549.1 |
