# Supplementary material for: Rickettsia parkeri hypothetical protein RPATATE_1266, a homolog of exopolyphosphatase/guanosine pentaphosphate phosphohydrolase, regulates tick cell apoptosis
Source: Microbiol Spectr. 2025 Jul 7;13(8):e00151-25. doi: 10.1128/spectrum.00151-25 (PMC12323366; doi:10.1128/spectrum.00151-25)
Supplement: Table S2 — Primers related to Methods. [file spectrum.00151-25-s0007.docx]

| Gene name | Forward primer (5’–3’) | Reverse primer (5’–3’) |
| --- | --- | --- |
| *caspase1*  *caspase3*  *cytochrome c* | AAACAAAGGACAACGCCGAC  AGTGAAAGGAGGGGTACCGA  AGAGCAGCCATGGTCGAAAT | GTCCTTGTAATCCGTGCCGT  CTCAGGATGCAGCAGACGAA  TCTGTGTAGCTGAAGCCTGC |
| *bcl-2* | TTTGCTCGCATACCGAGGTC | TACGACGAGATGCACGCCACA |
| *iap*  *gapdh*  *gltA (rickettsia)*  *tatC* | AACTCCCACTTGAAGATGGC  ATTGGAGACACCCACAGCAG TCGCAAATGTTCACGGTACTTT  TACCACGGCAATGCATCTT | CATGGTCGGAGACACCTGG  GACACGCTTCACTGGTCCTT TCGTGCATTTCTTTCCATTGTG  TTTGGAGTCGCCTTTCAACT |
| *folE* | ACGCCACGCATTGACATA | ATGACGGTACAGATAGCAGAAAG |
| *yihA* | CCACTGCTCTTTGACTGATATAGG | GGGCGTACTAGGCAAATCAA |
| *Rho* | CTTTACCTGATGACGGCACTAC | GCAGCTTGCAGAAATGGTTATC |
| *trxA* | CCGTATTCTGAAGGAGTGTTAGG | GGTGCGGACCGTGTAAA |
| *trxB* | ATGCTCTCCGCGTCATATTC | TGTCGGTACGGAAATTGTTAGT |
| *rpsD* | GCTGCAAGACAGTTAGTTTCAC | TCTATAACATCGCCTGCCTTTAG |
| *metG* | GAACTTACGAGGGACGGTTTAG | CTCAAGCAATTTATCCTGCCATT |
| *relA*(RPATATE_RS01890*) | AGTGGAAGTAGAGCAGCATTTAT | CCCGATCGAAGTAGCGATTATG |
| *ppx-gppa(Full length)* | GGACCTGACGCTAATCACTAAAATTATACCTAATATTTTTTCTAGC | CTCACGTAGATGCGTTCAGCTATTATTGATATCGG |
| *ppx-gppa(Insert Confirmation)***  *Plasmid specific primer**** | GGTATTCGGCAGGATACTTTATACGAAGTTATCAG  GGCGAATTCGAGCTCGGTA | AGATCTATAAGGCGAGATCACCAAGGTA  GGGCAATACCTAGCATAAGACGC |

**Supplementary Table S2. Primers, related to Methods.**

*bifunctional (p)ppGpp synthetase/guanosine-3',5'-bis(diphosphate)3'-pyrophosphohydrolase

** primers for identification of flanking region near insert at 5” and 3” end for single insertion confirmation.

*** Confirmation of shuttle vector transformation using plasmid-specific primers for the restoration of the ppx/gppa gene.
