# Supplementary material for: Rickettsia parkeri hypothetical protein RPATATE_1266, a homolog of exopolyphosphatase/guanosine pentaphosphate phosphohydrolase, regulates tick cell apoptosis
Source: Microbiol Spectr. 2025 Jul 7;13(8):e00151-25. doi: 10.1128/spectrum.00151-25 (PMC12323366; doi:10.1128/spectrum.00151-25)
Supplement: Table S3 — Predicted functional partner genes associated with ppx/gppa in six intracellular bacterial species and selected homologues in R. parkeri, related to Methods. [file spectrum.00151-25-s0008.docx]

**Supplementary Table 3.** Predicted functional partner genes associated with *ppx/gppa* in six intracellular bacterial species and selected homologues in *R. parkeri*, related to Methods.

| **Bacterial Species** | **Predicted Functional Partner Genes** | **Selected Homologues in R. parkeri** |
| --- | --- | --- |
| *Mycobacterium leprae* TN | *relA, eno, aroB* | *relA* |
| *Neisseria meningitidis* MC58 | *ppk, spoT, relA, nadK, ntpA, fbpA, metG* | *relA, metG* |
| *Salmonella enterica* subsp. *enterica* serovar Typhi CT18 | *relA, spoT, rpsD, tehB, tatE* | *relA, rpsD* |
| *Shigella boydii* BS512 | *relA, spoT, gppA, tehB, purM, purN, dsbC, tatE, surE* | *relA, tatC* |
| *Francisella tularensis* Biovar A str. SCHU S4 | *ppk2, relA, spoT, metG, rpsD, trxA1, guaB, ppnK, rho* | *relA, metG, rpsD, trxA, rho* |
| *Yersinia pestis* CO92 | *ppk, relA, spoT, pstA, ramA, tehB, pstb, pstC, pog, metG* | *relA, metG* |
